# CD4^+^ T_h_ immunogenicity of the *Ascaris spp*. secreted products

**DOI:** 10.1038/s41541-020-0171-z

**Published:** 2020-03-20

**Authors:** Friederike Ebner, Eliot Morrison, Miriam Bertazzon, Ankur Midha, Susanne Hartmann, Christian Freund, Miguel Álvaro-Benito

**Affiliations:** 1 Department of Veterinary Medicine, Institute of Immunology, Centre for Infection Medicine, Freie Universität Berlin, Robert-von-Ostertag-Str. 7-13, 14163 Berlin, Germany; 2 Laboratory of Protein Biochemistry, Department of Biology, Chemistry and Pharmacy, Freie Universität Berlin, Thielallee 63, 14195 Berlin, Germany

**Keywords:** Immunology, Diseases

## Abstract

*Ascaris spp*. is a major health problem of humans and animals alike, and understanding the immunogenicity of its antigens is required for developing urgently needed vaccines. The parasite-secreted products represent the most relevant, yet complex (>250 proteins) antigens of *Ascaris spp*. as defining the pathogen-host interplay. We applied an in vitro antigen processing system coupled to quantitative proteomics to identify potential CD4^+^ T_h_ cell epitopes in *Ascaris*-secreted products. This approach considerably restricts the theoretical list of epitopes using conventional CD4^+^ T_h_ cell epitope prediction tools. We demonstrate the specificity and utility of our approach on two sets of candidate lists, allowing us identifying hits excluded by either one or both computational methods. More importantly, one of the candidates identified experimentally, clearly demonstrates the presence of pathogen-reactive T cells in healthy human individuals against these antigens. Thus, our work pipeline identifies the first human T cell epitope against *Ascaris spp*. and represents an easily adaptable platform for characterization of complex antigens, in particular for those pathogens that are not easily amenable for in vivo experimental validation.

## Introduction

*Ascaris spp*. infections currently affect around 820 million people leading to impaired growth, impaired physical fitness and cognition, while also reducing general performance, in particular in children^1^. Considering the worldwide prevalence and intensity of Ascariasis in humans, it is critical to overcome the current limitations in controlling this parasitic infection. Improvements on infrastructure and educational programs together with revised mass drug administration programs will definitively contribute to mitigate the impact of Ascariasis^1^. However, the ideal solution would be the development of vaccines that prevents commonly observed re-infection after chemotherapy^2,3^. Candidate vaccines to prevent *Ascaris spp*. infection should be able to trigger effective antibody responses targeting essential antigens for the parasite to complete its life cycle^4^. The challenge is that these large, multicellular and cuticularized parasites confront the host with a complex mixture of protein antigens from which we have still very poor knowledge on their importance for infection or their immunogenicity. *Ascaris spp*. actively excrete and secrete complex mixtures of molecules, excretory secretory (ES) products, which are essential in parasite’s communication with its host, and shaping the host immune response^5–7^. The ES proteins comprise critical targets for vaccination in animal models and are expected to bear targets for vaccination in humans as well (reviewed in ref. ^2^). Interestingly, animal models have clearly shown the dependency on MHCII-restriction for the target of the antibody responses^8–10^. Thus, the deterministic nature of T-B cell responses—which implies that CD4^+^ T_h_ cell epitopes define the target of the antibody response—should contribute to define targets for the design of candidate subunit vaccines^11^. Therefore, identifying the epitopes that are involved in the host’s natural CD4^+^ T_h_ cell responses is essential to understand, monitor or modulate adaptive immune mechanisms that orchestrate *Ascaris spp*. expulsion.

CD4^+^ T_h_ cells recognize antigenic peptides presented by the major histocompatibility complex class II (MHCII) proteins expressed on antigen-presenting cells (APC). Peptides from antigens only become immunogenic when they are selected for presentation, and remain bound to MHCII molecules for a sufficient time to allow T cell surveillance. Thus, the abundance of antigens, their resistance to degradation and the affinity for the MHCII will define the potential immunogenicity of any peptide. To date, conventional in silico approaches predict peptide-MHC (pMHC) affinity mostly based on MHC pocket occupation by the peptide amino acids^12,13^, which is usually not very accurate for MHCII. Indeed, the IEDB CD4^+^ T cell immunogenicity prediction tool only reaches 50% of the immune response for those peptides below the percentile 20. However, these approaches ignore relevant aspects affecting epitope selection, such as the dynamics of peptide-MHCIIs^14,15^ (pMHCII), the peptide-editing function of HLA-DM^16^, and the influence of proteolytic activities on antigen presentation^17^. Although integrating proteolytic degradation improves the current conventional methods^18^, a robust in silico ranking of the most effectively presented peptides is still elusive. Experimental approaches based on recombinant proteins or subcellular fractions containing endosomal compartments rich on MHCII have been applied to define epitope selection on single antigens^19,20^. Culture of primary DC and quantitative immunopeptidomics of infected cells has also been used to define CD4^+^ T_h_ cell epitopes from *Listeria monocytogenes* in mouse^21^. However, the complex infection cycle of *Ascaris* spp. and of its antigens pose a considerable challenge for any of these methods. Consequently, to date, there is no experimental set-up described to define the CD4^+^ T_h_ immunogenicity of complex antigenic mixtures as nematode ES.

## Results and discussion

During infection *Ascaris* parasite*s* confront the host with a mixture of the ES from female (ESF) and male (ESM) worms, which are considered to bear candidates for subunit vaccines^22^. Parasitic nematodes are suggested to be polygamous and often show female-biased sex ratio within the distribution in a host. Analyzing female and male ES antigens separately for their influence on CD4^+^ T_h_ cell responses presents an unbiased approach to account for pathogen gender-heterogeneity during infection, sexual dimorphism (e.g., size) and gender-associated genes/proteins as reported previously for other parasitic nematodes^6,7,23,24^.

**Fig. 1:**
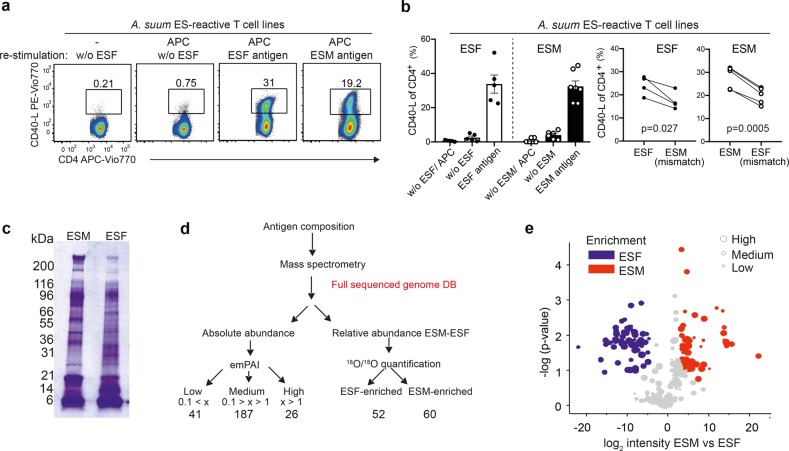
CD4^+^ T_h_ cell activation in *Ascaris* ES antigen-specific T cell lines and ES antigen composition. **a** For generating *Ascaris suum* ES-specific T cell lines, PBMCs from healthy donors were stimulated with 40 µg/mL ES antigen for 6 h, enriched for CD40-L^+^ cells and expanded for 2 weeks (see Supplementary Fig. 1a). Expanded ES-reactive T cells were re-stimulated with or without (w/o) ESF or ESM-antigen-primed, CD3-depleted APC and percentages of CD40-L^+^ antigen-reactive T cells among CD4^+^ cells are indicated above gates. **b** Percentages of CD40-L^+^ antigen-reactive T cells among *A. suum* ES-reactive T cell lines re-stimulated with ESF or ESM antigen, or with mismatched ES antigens for *n* = 3–5 different donors (left: mean with SEM, right: paired *t*-test). **c** Model antigenic mixtures derived from *Ascaris* ES products are different in protein composition. SDS-PAGE of ES male (ESM) and ES female (ESF) mixtures (40 μg of antigen loaded per well). **d** A mass-spectrometry-based approach used to determine composition of male and female ES products. The emPAI and the ESF *vs*. ESM (^16^O/^18^O) ratios are defined after tryptic digest defining the total abundance and the differences between ESF and ESM. **e** Volcano plot showing differences in protein composition determined by mass spectrometric analysis. The depicted log_2_-fold intensity difference (ESM vs. ESF) represents the difference in geometric mean values from ^18^O labeling. On the *y* axis the –log (*p*-value) for the difference observed is indicated. Blue symbols indicate ESF-enriched and red corresponds to ESM-enriched. The different size of the corresponding symbols indicates the determined emPAI value as shown in the legend.

We generated human T cell lines from healthy volunteers reacting to ESF or ESM antigens using the antigen-specific T cell enrichment and expansion as described by Bacher et al.^25^ (Supplementary Fig. 1a). This approach helped to overcome the expected low in vivo frequency of any potential *Ascaris* ES-specific CD4^+^ T_h_ cells in healthy (uninfected) donors. The presence of reactive T cells and its low frequency was confirmed by CD40-L staining (Supplementary Fig. 1b). CD40-L, is specifically expressed by CD4^+^ T_h_ cells shortly after TcR-mediated antigen recognition irrespectively of the restricting MHC allele and can be used to assess and enrich antigen-specific T cells^26^. Re-stimulation of the generated cell lines specific for ES antigens resulted in a remarkable increase on CD40-L^+^ cells when compared to the corresponding controls (Fig. 1a). Upregulation of CD40-L and CD40-L/cytokine co-expression (Supplementary Fig. 1c) after re-stimulation confirms a functional CD4^+^ T_h_ phenotype of *Ascaris*-reactive T cells. Interestingly, when testing reactivity of T cell lines for the mismatched ES antigen we could detect a lower proportion of reactive T cells, suggesting the presence of gender specific-T cell responses (Fig. 1b).

The observed gender-specific T cell responses motivated us to perform a proteomic characterization of ESF and ESM antigens^6,7^ (Fig. 1c–e). Our aim was to identify ESF and ESM antigens bearing potential CD4^+^ T_h_ epitopes. By combining the use of the exponentially modified Protein Abundance Index (emPAI) with ^16^O/^18^O-labeling (Fig. 1d) we defined the ESM and ESF composition, thereby retrieving absolute protein abundances of ESM and ESF extracts (Fig. 1e), and the relative difference between them (Fig. 1e). This analysis yielded additional information regarding the differences in abundance between ESF and ESM (Supplementary Table 1) in comparison to the previously described dataset^7^. The difference in the number of protein sources found here when compared with the previous study (175 previously vs. 254 in our case) arises from our conservative approach when considering the source of tryptic peptides. Rather than selecting a single leading protein, we explicitly kept protein entries with small differences in their primary sequences but which are not clearly distinguishable by conventional shotgun MS. This characterization could be used either as a reduced sampling space for potential CD4^+^ T_h_ cell epitopes *vs*. the whole *Ascaris spp*. predicted proteome, or as an internal control for further experiments in which these 250 proteins are expected to be the immunogenic determinants of host responses to *Ascaris spp*. We found large differences in the abundance of proteins either within a single extract or between ESM and ESF (fold-differences up to 10^3^ for emPAI and 10^7^, respectively).

The identification of CD4^+^ Th cell epitopes from the ES products, with 1.5 × 10^5^ potential 15-mers, represents a challenge. In silico, one could make use of binding predicting tools such as NetMHCIIPan reducing the number of candidates since only strong and weak affinity binders are expected to be immunogenic. For a set of seven common MHCII allotypes NetMHCIIPan defines between 1.2 × 10^4^ to 3.9 × 10^4^ peptides for the ES (Supplementary Fig. 2a). More sophisticated approaches as the IEDB CD4 T cell immunogenicity prediction tool^27^ (IEDBcd4) rely on a combination of binding affinity prediction, and neural network trained on experimentally validated epitopes from the IEDB. Thus, candidates ranked in the first 20th percentile would bear around half of the immunogenic candidates of an antigen. In this case, the number of CD4^+^ T_h_ cell epitope candidates within the ES lies in the range of 800–1500 for the same MHCII allotypes (Supplementary Fig. 2b). However, these methods ignore relative and total protein abundances that could be of relevance when assessing the immunogenicity of ESF and ESM. Experimentally, the most popular approaches rely on the identification of pathogen-derived MHCII-bound peptides by MS. Either cell cultures or animal models infected with the pathogen, or provided with the corresponding antigens, are the sources for isolation of MHCII-peptides. The availability of adequate infection models and the lack of biological replicating capacity of the ES poses a major challenge for any of such approaches. We hypothesized that an in vitro experimental approach recapitulating key events of antigen presentation such as proteolysis of antigens by cathepsins and catalyzed peptide exchange by HLA-DM, should contribute to define potential CD4^+^ T_h_ epitopes. We reasoned that this minimalist experimental approach described by Sadegh-Nasseri’s group^19^ should facilitate the identification of potential epitopes when using complex antigenic mixtures. Indeed, using this system, the lower background of self-peptides will benefit the MS identification of pathogen derived antigenic peptides when compared to mass spectrometry analysis of MHCII-associated peptidomes from cell culture or in vivo samples.

**Fig. 2:**
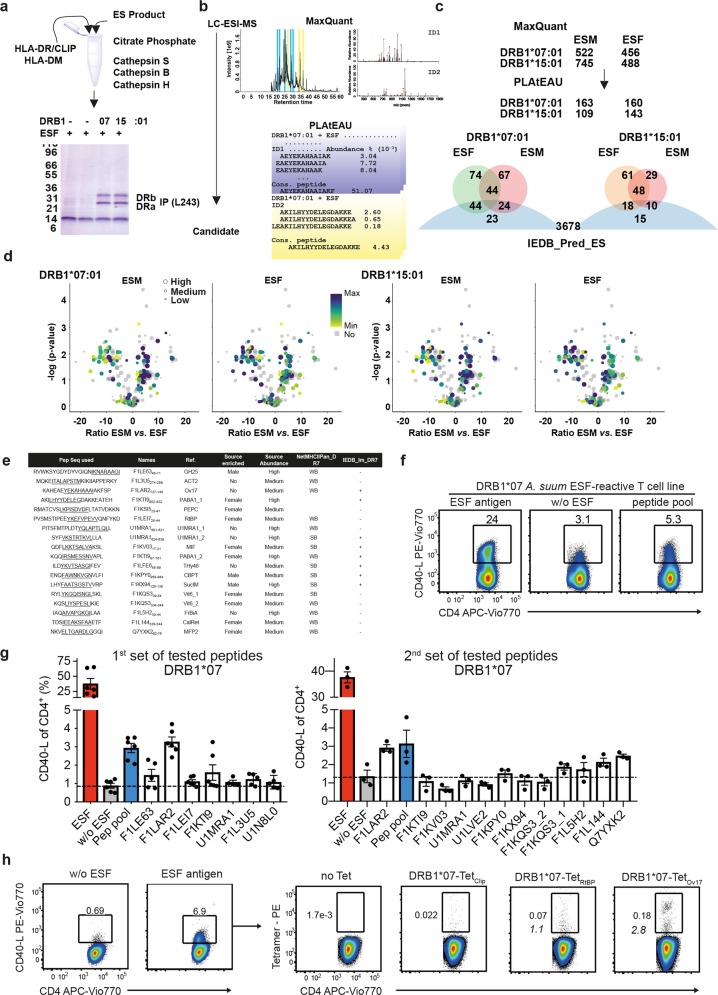
The use of a reconstituted in vitro antigen processing facilitates the detection of CD4^+^ T_h_ cell epitopes. **a** ES products are incubated in vitro with recombinant proteins and adequate buffer conditions. Proteolytic activities used degrade most of the components of the reactions except for MHCII proteins which are subsequently pull down using a conformation specific antibody (L243). **b** MaxQuant is used for their identification and PLAtEAU defines series of nested peptides and retrieve the consensus peptides and corresponding MS1 intensities according to the MaxQuant output. For each peptide a relative abundance value is retrieved based on the MS1 intensity and the total ion current from the run. This approach yields a list of candidate epitopes with relative abundance values and predicted affinities. **c** Summary of the performance of the experimental determination of candidate antigens for each condition tested. The overlap between the peptide sets (based on predicted binding cores to facilitate comparisons) for each allotype and ES antigen, and the predicted IEDB immunogenicity score is shown as Venn Diagrams (sized according to numbers, the IEDB set consists of 3678 entries and it is cut in this figure but shown in full in Supplementary Fig. 2f). **d** Mapping of the identified potential epitopes to their corresponding protein sources using the intensity color code shown in the legend. **e** Summary of peptides used to evaluate the performance of the reconstituted in vitro system. The peptide sequence is shown in the first column and underlined is shown the binding core predicted for DRB1*07:01. The corresponding protein source with the amino acid positions covered by the peptide are indicated in the second column. Abbreviated uniprot names are provided. Last columns include the relative and total abundance of each protein sources and the predicted binding affinity for DRB1*07:01 and whether any peptide with the same binding core (underlined) is predicted to be immunogenic by IEDBcd4 prediction tool. **f** Representative dot blot example for a ESF antigen-specific T cell line generated from an healthy DRB1*07-typed volunteer and re-stimulated with either whole ES antigen (40 µg/mL), no antigen (w/o) or a pool of synthetic peptides (25 µg/mL for each peptide) selected from the in vitro reconstituted HLA-DRB1*07:01 experimental data set (**f**). **g** Summarizes CD40-L frequencies among CD4^+^, indicative for peptide recognition by CD4^+^ T cells, for whole ES antigen, peptide pool and single peptide re-stimulations. Peptide sequences are indicated in Table (**f**). Combined are data from the same healthy, DRB1*07-typed volunteer from *n* = 3 separate experiments with *n* = 2 separate re-stimulations (1st peptide set) or *n* = 1 experiment with *n* = 3 separate re-stimulations (2nd peptide set). CD40-L frequencies per experiment were corrected for individual background CD40-L expression of w/o antigen/w/o APC controls (mean with SEM). **h** Representative dot blots of an *Ascaris* ESF-specific, DRB1*07T cell line analyzed for *Ascaris* ESF peptide-specific tetramer staining. Left side indicates overall frequency of ESF antigen specific CD4^+^ cells after expansion compared to control. Right side shows corresponding tetramer staining with DRB1*07:01-Tet-_CLIP_ (control), Tet-_RtBP_ and Tet_Ov17_ gated on CD4^+^ T cells after expansion. Italic numbers indicate calculated Tet^+^ frequency relative to proportion of ESF antigen-specific T cells.

We used for our experiments the ES antigens (ESM or ESF), two common MHCII allotypes (DRB1*07:01 or DRB1*15:01, Supplementary Fig. 2c) preloaded with the placeholder Class II invariant chain peptide-CLIP and HLA-DM, which functions as peptide-editor^16^ (details in Supplementary Fig. 2d) and the commercial proteases previously described^19^ (Fig. 2a). Recombinant MHCII proteins are resistant to proteases (Fig. 2a) and can be further pulled-down from the mixtures to elute and determine the bound peptides by LC-ESI-MS. We used MaxQuant for peptide identification considering a customized database that includes all the potential entries derived from the Ascaris genome, and the molecules of the in vitro reconstitution system (MHCII, HLA-DM and proteases) (Fig. 2b). As expected, the majority (>99.0%) of the peptides belong to protein sources identified in the ES. The information on MS1 intensity for each identified peptide was used by our recently described epitope analysis tool, PLAtEAU^28^, to define consensus peptides including their relative abundance for each series of peptides that contain a common core but vary in the length of their N-terminal and C-terminal extensions (Fig. 2b). This experimental workflow dramatically reduces the number of potential CD4^+^ T_h_ epitopes to less than 10^3^ peptides for all conditions, which once analyzed with PLAtEAU resulted in around 150 candidate epitopes for each condition (details given in Fig. 2c and Supplementary Fig. 2e). The full list of peptide identified consists of a total of 335 potential T cell epitopes, which were annotated based on the abundance of the corresponding protein source, the predicted affinity for each allotype, and whether the binding core is found in any peptide defined by the IEDBcd4 (Supplementary Table 2). There is a considerable overlap between the consensus peptides selected by each allotype for the ESF and ESM, and around one third are found in the lists of IEDBcd4 predicted immunogenic peptides (Fig. 2c and Supplementary Fig. 2f). More interestingly, there are certain peptides found exclusively enriched in for either ESF or ESM. The presence of predicted weak and high affinity binders increases from 10 and 1% in the pool of all potential peptides of the ES to 20 and 7% in the experimentally determined peptides, respectively. The presence of peptides with no predicted affinity for the MHCII allotypes used may represent mainly intermediate steps of the antigen processing reactions that are stable enough to overcome the immunoprecipitation process and that would be exchanged under more dynamic conditions of protein turnover in the living cell. Thus, the protein concentrations available under the test-tube conditions assayed may represent a limiting factor. Hierarchical clustering analysis of the annotated potential epitopes based on their abundance reveals that both MHCII molecules selected mostly epitopes from proteins with intermediate and high emPAI values, and in particular from those enriched (e.g., ESF:ESM ratio > 2) in the respective antigen source (ESM or ESF) (Volcano plots in Fig. 2d and Supplementary Fig. 2g).

We selected a limited set of peptides that would allow us to test the performance of the reconstituted in vitro system on its own and in comparison to in silico prediction tools to define immunogenic candidates (Figure 2e). We initially selected a limited set of six candidates including the Ov17 (F1LAR2_127–146_) consensus peptide defined exclusively under DRB1*07:01 + ESF conditions (predicted to be immunogenic by IEDBcd4 but with weak affinity for the restricting allele). This peptide represents an ideal candidate to prove the selectivity and performance of our experimental approach. Experiments on swine and mouse models have shown the potential of the OV17 antigen (F1LAR2/As16) for conferring protection to *Ascaris spp*. infection^29–31^, and furthermore the restricting allele reaches up to 9% of the global population and even higher frequencies in *Ascaris spp*. endemic areas (up to 10.5% in Africa and 15% in Asia)^32^. Other peptides included in this list comprise candidates excluded by either the in silico or experimental approaches. We queried whether the TcR pool present in healthy individuals would respond to these candidates. We derived ESF T cell lines from a healthy, DRB1*07-typed volunteer and assessed CD40-L expression after re-stimulation with either whole antigen, the selected pool of six peptides, or single peptide loaded APCs (Fig. 2f). Strikingly, we found that OV17, and to a lower extent PABA1_1 peptides, which we selected as potential CD4^+^ T_h_ cell epitopes experimentally and also considered as potentially immunogenic by IEDBcd4 yield a T cell response above the background (dashed line) (Fig. 2g). We further verified the specificity of the experimental approach by testing this same set of peptides using ESF expanded cell lines from a DRB1*03:01; DRB1*15:01 volunteer (Supplementary Fig. 2h). We additionally tested a second set of peptides including candidates found by our experimental approach and neglected by either one or both in silico tools (Fig. 2e). Interestingly, we confirm the immunogenicity of four candidates excluded by the IEDBcd4 and with weak affinity prediction for DRB1*07:01 (Fig. 2e, g). Note that again in these experiments we re-confirm the Ov17 peptide as a prime candidate of immunogenicity in the DRB1:07:01 background. In summary, the proposed experimental approach outperforms to the use of either prediction tools on their own.

Considering the co-dominant expression of other HLA genes in the donor-derived cell lines, the reactivity observed could arise from the presentation of the corresponding peptides by any MHCII allotype present in the APCs. We confirmed the DRB1*07:01 restriction for the presentation of the OV17 peptide by tetramer staining of ESF CD4^+^ T cell lines. A significant pool of CD4^+^ T cells responding to this peptide is detected when it is displayed by DRB1*07:01 tetramers compared to control tetramers (DR7_CLIP_ or DR7_RtBP_ tetramers; Fig. 2h and Supplementary Fig. 1e). Of note, the overall low frequency of DRB1*07:01-Tet_Ov17_^+^ cells can be explained by the low frequency of whole antigen-reactive T cells for the cell line applied in that assay (only 6.9% CD40-L^+^, Fig. 2g), but still reflect that 2.8% of all ESF reactive T cells bind DRB1*07:01-Tet_Ov17_.

Together, we demonstrate that a reduced set of in vitro and ex vivo experiments is extremely useful to define human CD4^+^ T_h_ cell epitopes from complex antigenic mixtures bypassing the need of animal models^33^ or immunization in humans^34^. The use of controlled redox potential and/or the gamma-interferon-inducible lysosomal thiol reductase^35^ should further contribute to improving the presented approach by facilitating access to disulfide-bonded epitopes. However, presentation of the selected candidates already represents an excellent platform for testing the importance of specific antigen processing factors such as HLA-DM^36^ or its competitive inhibitor HLA-DO^37^. Conditions can be tuned to include distinct MHC allotypes or combinations thereof, and the nature of the antigen can reach from single proteins to complex mixtures derived from secretomes, complete pathogens or cellular lysates. From a biological point of view we characterize the OV17 (F1LAR2_127–146_) epitope as a human CD4^+^ T_h_ cell epitope for *Ascaris spp*. and show its restriction by the DRB1*07 allotype. Immunization with this antigen in animal trials elicits considerable protective immunity to *Ascaris spp*. including specific antibodies and CD4^+^ T_h_ cells^29–31^. It will thus be of great importance to further characterize immune responses against the OV17/AS16 peptide in either carriers or non-carriers of DRB1*07:01 to confirm the potential of these antigen for vaccination. Another interesting candidate is PABA1^10^, showing a completely different peptide selection pattern for the two alleles used, with a considerably higher number of peptides selected by DRB1*15:01 when compared to DRB1*07:01 (7 vs. 1 respectively). Prospectively, the stage is then set to investigate a pooled sample of a limited number of antigens to profile the T cell immune status of infected individuals. Furthermore, gaining access to HLA-typed material will contribute to define the deterministic nature of B-T cell responses to complex antigens to rationalize the development of vaccine subunits to *Ascaris* spp.

## Methods

### Antigen preparation

Excretory-secretory (ES) antigens were prepared from worm culture supernatants of male and female adult *Ascaris* spp. worms obtained from a local slaughter house. In brief, worms were separated by sex and washed several times in a balanced salt solution (BSS) containing antibiotics and used as culture media for adult worms (127 mM NaCl, 7.5 mM NaHCO_3_, 5 mM KCl, 1 mM CaCl_2_, 1 mM MgCl_2_, 200 U/mL penicillin, 200 μg/mL streptomycin, 50 μg/mL gentamicin, 2.5 μg/mL amphotericin B) and kept at 37 °C with 5% CO_2_. Media was replaced on a daily basis, sterile filtered through a 0.22 μM vacuum-driven filter system and collected for ES antigen preparations starting 48 h after beginning of worm culture and finally stored at −20 °C until further use. Worm culture supernatants collected over 1 week were further concentrated using centrifugal protein concentrators with a 5 kDa MWCO (Vivaspin, Sartorius, Göttingen, Germany) to obtain the final, concentrated ESF antigen (from female worms) and ESM (from male worms).

### Mass spectrometry

Peptide mixtures were analyzed by a reversed-phase capillary system (Ultimate 3000 nanoLC) connected to an Orbitrap Velos (Thermo Fischer) using conditions and settings described in the ref. ^28^. In brief, peptides reconstituted in 0.1% (v/v) TFA, 5% (v/v) acetonitrile, and 6.5 µL were loaded into a reversed-phase capillary nano liquid chromatography system (Ultimate 3000, Thermo Scientific, USA) connected to an Orbitrap Velos mass spectrometer (Thermo Scientific). LC separation was performed on a capillary column (Acclaim PepMap100 C18, 2 μm, 100 Å, 75 μm i.d. × 25 cm, Thermo Scientific) at an eluent flow rate of 300 nL/min. Mobile phase A contained 0.1% formic acid in water, and mobile phase B contained 0.1% formic acid in acetonitrile. The column was pre-equilibrated with 3% mobile phase B followed by a linear increase up to 50% of mobile phase B in 50 min. Mass spectra were acquired in a data-dependent mode utilizing a single MS survey scan (*m*/*z* 350–1500) with a resolution of 60,000 in the Orbitrap, and MS/MS scans of the 20 most intense precursor ions in the linear trap quadrupole.

For quantitative proteomics, we used ^16^O/^18^O-labeled samples. Male and Female ES products (ESM and ESF, 50 μg) were resolved in SDS-PAGE (4–20%). Each lane was cut into ten pieces of equal size in a parallel fashion. In gel tryptic digestion and ^16^O/^18^O labeling was performed as following a protocol as described in^38^. Briefly, gel bands were incubated with 50 ng trypsin in 15 μL 50 mM ammonium bicarbonate buffer in the presence of heavy (H_2_^18^O) and light (H_2_^16^O) water overnight at 37 °C. Residual trypsin activity was inactivated by adding 10 μL of 0.5% TFA in acetonitrile. Matching heavy and light samples from either Male and Female ES were mixed before drying the samples in a Speed-Vac. Samples were reconstituted in 10 μL 0.1% (v/v) TFA and 5% (v/v) acetonitrile before measurements. Peptide identification was performed using Mascot Distiller (version 2.4.3.3) software. Data were searched against the Uniprot *Ascaris* spp. protein database (March 2017). The mass tolerance of precursor and sequence ions was set to 10 ppm and 0.35 Da, respectively. For quantification, only unique peptides were used.

Peptides eluted from the reconstituted in vitro antigen processing system were measured as described and MaxQuant software (version 1.5.2.8) was used for peptide identification. Customized databases featuring reviewed and non-redundant Uniprot *Ascaris* spp. proteins from uniprot were used (accessed March 2017) for the in vitro reconstituted experiments to which we included all other recombinant proteins used in the assay, namely human cathepsins, HLA-DR2 and HLA-DR7, as well as HLA-DM. No enzyme specificity was used for the search, and a tolerance of 10 ppm was allowed for the main ion search and 50 ppm for the MSMS identification The “match between runs” feature was enabled. The FDR was set at 0.01 (1%). Reverse IDs and known contaminants like keratins were filtered before further data analysis.

NetMHCIIPan 3.2 was used to predict peptide binding to the indicated HLA-DR allotypes. The protein database generated upon the MS analysis (only quantified proteins) was loaded into the webserver using a 2 and 10% cutoff for strong and weak binders (unless otherwise indicated).

### Constructs, protein expression, and purification

DNA constructs encoding HLA molecules used in this study have been generated according to the sequences available in the IMGT/HLA database (http://www.ebi.ac.uk/ipd/imgt/hla/). The cDNAs encoding the different HLA subunits were cloned into the pFastBacDual. Recombinant proteins were expressed in Sf9 cells infected at an MOI of 5 for 72 h. Supernatants were concentrated and dialyzed (in PBS) using a Vivaflow200 tangential filter. To purify the target proteins by immunoaffinity chromatography the concentrated and buffer exchanged concentrates were applied to either an anti-HLA-DR-FF-sepharose or M2 anti flag (Sigma)^16^ for HLA-DR and HLA-DM, respectively.

Depending on the application, HLA-DR molecules were further treated prior to their use with only Thrombin (20 U/mg protein; for tetramer preparation) or with Thrombin and V8 protease (10 U/mg; in vitro reconstituted system) for 2 h at 37 °C. Subsequently proteases were inactivated by adding Complete protease inhibitor cocktail (Roche) and further gel-filtrated, while HLA-DM proteins were directly subjected to gel filtration. Both types of HLA proteins were gel-filtrated in a Sephadex S200. Fractions containing the proteins of interest were pooled and concentrated with Vivaspin 10 KDa MWCO spin filter.

### Peptide selection and synthesis

The complete list of peptides obtained after PLAtEAU analysis for all experiments was loaded into excel as a single list. We used the excel random selection function to generate a list of four peptides from the whole dataset. The corresponding sets of four peptides (500 iterations) were screened to define those with two peptides originating from the same antigen (ESF or ESM) and found in one set of experiments (DRB*01:07 or DRB*15:01). An additional criteria included the presence on the set of four peptides of at least one peptide not found in the corresponding experiment (used as control). The corresponding list of peptides indicated arises as the one fulfilling these criteria and having the larger distance between pIm values. Synthetic peptides were subsequently purchased from Peptides and Elephants (Berlin, Germany). Purity as stated by the vendor was more than 95%. All peptides were protected in their N-termini and C-termini by addition of an Ac and NH_2_ group, respectively.

### In vitro reconstituted antigen processing system

The cell-free reconstituted in vitro system described by Sadegh-Nasseri et al.^19^ was modified according to the specific needs of the experiments. HLA molecules (1 μM) together with the candidate antigens (200 μg/mL) and HLA-DM (0.25 μM) were incubated for 2 h at 37 °C in citrate phosphate 50 mM pH 5.2 in the presence of 150 mM NaCl. Cathepsins were added to reaction mixtures after incubation with l-Cysteine (6 mM) and EDTA (5 mM). Cathepsin B (Enzo), H (Enzo), L (Enzo), and S (Sigma) were used for our in vitro experiments at molar ratios (cathepsin:substrate) ranging from 1:250 to 1:500. The final reaction mixture was incubated at 37 °C for 2–5 h. Afterwards the pH was adjusted to 7.5 and Iodoacetamide was added (25 μM). Immunoprecipitation (IP) of the pMHCII complexes was performed using L243 covalently linked to Fast Flow sepharose. Peptides were eluted from purified MHCII adding TFA 0.1% to the samples. Peptides were separated from the MHCII molecules by using Vivaspin filters (10 kDa MWCO) and a subsequent reverse phase C18 enrichment. The filtrates were further lyophilized and resuspended for mass spectrometry analyses in a mixture of H_2_O (0.94):AcN (0.05):TFA (0.01).

### Cell isolation and stimulation

Permission for working on human primary cells was obtained for the study “Functional characterization of human *Ascaris* spp. specific CD4^+^ T cells“ (ethic committee of Charité—Universitätsmedizin Berlin, EA1/190/16). DRB1 background-typed PBMC were obtained from healthy lab volunteers. Peripheral blood mononuclear cells (PBMC) were isolated by density gradient centrifugation over human Pancoll (1.077 g/mL, PAN-Biotech, Aidenbach, Germany) and rested over-night in serum-free RPMI-1640 supplemented with 100 U/mL penicillin and 100 µg/mL streptomycin (all from PAN-Biotech, Aidenbach, Germany). For enrichment, 2 × 10^7^ PBMC/mL/well were stimulated for 6 h with excretory-secretory antigen mixtures of female *Ascaris* spp. worms (ES antigen F, 40 µg/mL) in the presence of CD40 (clone HB14, 1 µg/mL functional grade pure, #130-094-133, Miltenyi Biotec, Bergisch Gladbach, Germany) and CD28 (clone CD28.2, 1 µg/mL, #555725, BD Pharmingen^TM^, NJ, US). Following stimulation, antigen-specific T cells were separated using the CD154 MicroBead Kit (#130-092-658, Miltenyi Biotec, Bergisch Gladbach, Germany) according to the manufacturer’s suggestions. That included labeling first with CD154-biotin and magnetically anti-biotin microbeads in a second step followed by enrichment using MS MACS columns (Miltenyi Biotec, Bergisch Gladbach, Germany).

For assessing precursor frequencies of *Ascaris* ES antigen F specific CD4^+^ T cells, PBMC were stimulated as described above and Brefeldin A (3 µg/mL, Thermo Fisher Scientific) was added after the first 2 h of stimulation.

### Generation of *Ascarissuum* ES antigen-specific T cell lines and restimulation

The generation of Ag-specific T cell lines was performed as described by Bacher et al.^25^. In brief, the stimulated and isolated CD154^+^ *Ascaris* ES antigen F-specific T cells were cultured 1:100 with autologous, mitomycin C (Sigma-Aldrich) treated feeder cells in X-VIVO™ 15 (Lonza, Basel, Switzlerland) supplemented with 5% human AB serum (PAN-Biotech, Aidenbach, Germany), 100 U/mL penicillin, 100 μg/mL streptomycin (PAN-Biotech, Aidenbach, Germany), and 50 ng/mL IL-2 (PeproTech, NJ, US). Cells were expanded for 14 days and culture medium was replenished with IL-2 containing media when needed. For restimulation after 14 days, autologous PBMC were CD3-depleted using BD FACSAria™ III cell sorter or CD3-bead MACS and co-cultured 1:1 with expanded T cell lines in the presence of the indicated antigens. For assessing the frequency of total *Ascaris* ES antigen F and/or M reactive T cells after expansion, co-cultured cells were restimulated with 40 µg/mL ES antigen F for 6 h. For addressing peptide specificities, restimulation was performed with single, synthetic peptides (25 µg/mL) or a pool of all peptides (25 µg/mL of each peptide). Brefeldin A (3 µg/mL) was added after the first 2 h of stimulation.

### Antibody staining and flow cytometric analysis

The following monoclonal antibodies reactive with human species were used for staining: CD8-VioGreen (BW135/80, cat.: 130-113-726, 1:50), CD4-APC-Vio770 (VIT4, cat.: 130-113-211, 1:50), CD154/CD40L-PE-Vio770 (5C8, cat.: 130-096-793, 1:10), CD20-VioGreen (LT20, cat.: 130-113-379, 1:50), CD14-VioGreen (TÜK4, 130-113-153, 1:50) (all from Miltenyi Biotec, Bergisch Gladbach, Germany);

IL-13-FITC (PVM13-1, cat.: 11-7139-42, 1:20 or 85BRD, cat.: 11-7136-42, 1:20), IL-4-PE (8D4-8, cat.: 12-7049-42, 1:20) (all from Thermo Fisher Scientific); TNFa-Pacific Blue (MAb11, cat.: 502920, 1:100), IFNg-PerCp-Cy5.5 (4S.B3, cat.: 502526, 1:20), CD3-APC (Okt3, cat.: 17-0037-42, 1:50) (all from Biolegend, San Diego, CA, US). Fixation and permeabilization were performed using the Foxp3 Transcription Factor Staining Buffer Set (Thermo Fisher Scientific) according to the manufacturer’s suggestions followed by intracellular cytokine and CD154/CD40-L staining. Fixable viability dye was used in eFluor-506 (cat.: 65-0866-14, 1:1000, Thermo Fisher Scientific).

Cells were acquired using BD FACSCanto II (with Diva software, Heidelberg, Germany) and post-acquisition data analysis was carried out using FlowJo software (TreeStar, Ashland, OR, US).

### Tetramer preparation and staining

Purified recombinantly expressed HLA molecules were treated with Thrombin and subsequently subjected to size exclusion chromatography (Sephadex S200). The placeholder peptide CLIP was exchanged by the indicated peptides incubating HLA molecules with 50 molar excess of the desired peptide for 72 h in the presence of molecular loading enhancers. In brief, the FR dipeptide (150 μM) and AdaEtOH (100 μM) were used to promote CLIP exchange for the corresponding peptides. After gel filtration the peptide loading of HLA-II complexes was verified by MS. The generated peptide HLA class II complexes were biotinylated in a BirA sequence (DRB chain) using a BirA ligase (Avidity). The Biot-peptide-HLA class II complexes were then used to generate tetramers using Streptavidin-PE. Tetrameric complexes were finally separated by gel filtration and stored in PBS + NaAz (0.02%).

For peptide-specific tetramer staining, expanded cells were incubated in sodium azide (5 mM) containing X-VIVO™ 15 (Lonza) supplemented with 5% human AB serum (PAN-Biotech, Aidenbach, Germany), 100 U/mL penicillin, 100 μg/mL streptomycin (PAN-Biotech, Aidenbach, Germany) for 2 h at 37 °C and 5% CO_2_ in the presence of the PE-SAv-Tetramers specific for CLIP, Ov17, or RtBp at final concentrations of 20 µg/mL. Following incubation, cells were washed and counterstained for CD8, CD4, CD20, CD14, and viability.

### Statistical analysis

GraphPad Prism 7.0 software (GraphPad Software San Diego CA, USA) was used in general for statistical analysis. Variance was calculated with the two-way ANOVA method. The null hypothesis was rejected when the *p* value was lower than 0.05.

Perseus software^39^ was mainly used to analyze the MS data. Epitopes identified by the PLAtEAU algorithm (% Intensity from the TIC) were loaded as matrixes into Perseus. Data was log2 transformed and missing values were imputed as 0. The resulting matrices were plotted as heat-maps. Columns were hierarchical clustered with “average” as agglomeration method and “Pearson correlation” as distance matrix. Rows were ordered by hierarchical clustering using “average” as agglomeration method and “euclidean” as distance matrix. Epitopes eluted from each experimental condition were grouped and used to define the mean intensity value for each peptide or epitope. *p* values were calculated based on the observed intensities using *t*-test, in this case an FDR of 0.01 and a S0 = of 2 were used.

### Reporting summary

Further information on experimental design is available in the Nature Research Reporting Summary linked to this article.

## Supplementary information

Supplementary Information

Reporting Summary

## Data Availability

The mass spectrometry proteomics data have been deposited to the ProteomeXchange Consortium (http://proteomecentral.proteomexchange.org) via the PRIDE partner repository^40^ with the dataset identifier PXD015924 and PXD015012. Constructs for the recombinant expression of MHCIIs are available upon request. The PLAtEAU algorithm can be retrieved from https://github.com/e-morrison/plateau, or used as a webtool at: https://plateau.bcp.fu-berlin.de/.
