# Supplementary material for: CD4+ Th immunogenicity of the Ascaris spp. secreted products
Source: NPJ Vaccines. 2020 Mar 20;5:25. doi: 10.1038/s41541-020-0171-z (PMC7083960; doi:10.1038/s41541-020-0171-z)
Supplement: Supplementary file 1 — Supplementary Information [file 41541_2020_171_MOESM1_ESM.pdf]

Supplemental Information

CD4+ T_h immunogenicity of the *Ascaris spp*. secreted products

Friederike Ebner[1*], Eliot Morrison[2], Miriam Bertazzon[2], Ankur Midha[1], Susanne Hartmann[1], Christian Freund[2], Miguel Álvaro-Benito[2*]

[1] Institute of Immunology, Centre for Infection Medicine, Department of Veterinary Medicine, Freie Universität Berlin, Robert-von-Ostertag-Str. 7-13, 14163 Berlin

[2] Laboratory of Protein Biochemistry, Department of Biology, Chemistry and Pharmacy, Freie Universität Berlin, Thielallee 63, 14195 Berlin

* Contributed equally

To whom correspondence should be addressed: Miguel Álvaro Benito or Christian Freund, Laboratory of Protein Biochemistry, Department of Biology, Chemistry and Pharmacy. Freie Universität Berlin, Thielallee 63, 14195 Berlin; or Susanne Hartmann, Institute of Immunology. Centre for Infection Medicine. Department of Veterinary Medicine. Freie Universität Berlin. Robert-von-Ostertag-Str. 7-13, 14163 Berlin

Telephone: +49 30 838 52288
email: malvaro@zedat.fu-berlin.de, chfreund@fu-berlinl.de, susanne.hartmann@fu-berlin.de

Supplementary Figures

Supplementary Tables

Supplementary Figure 1.

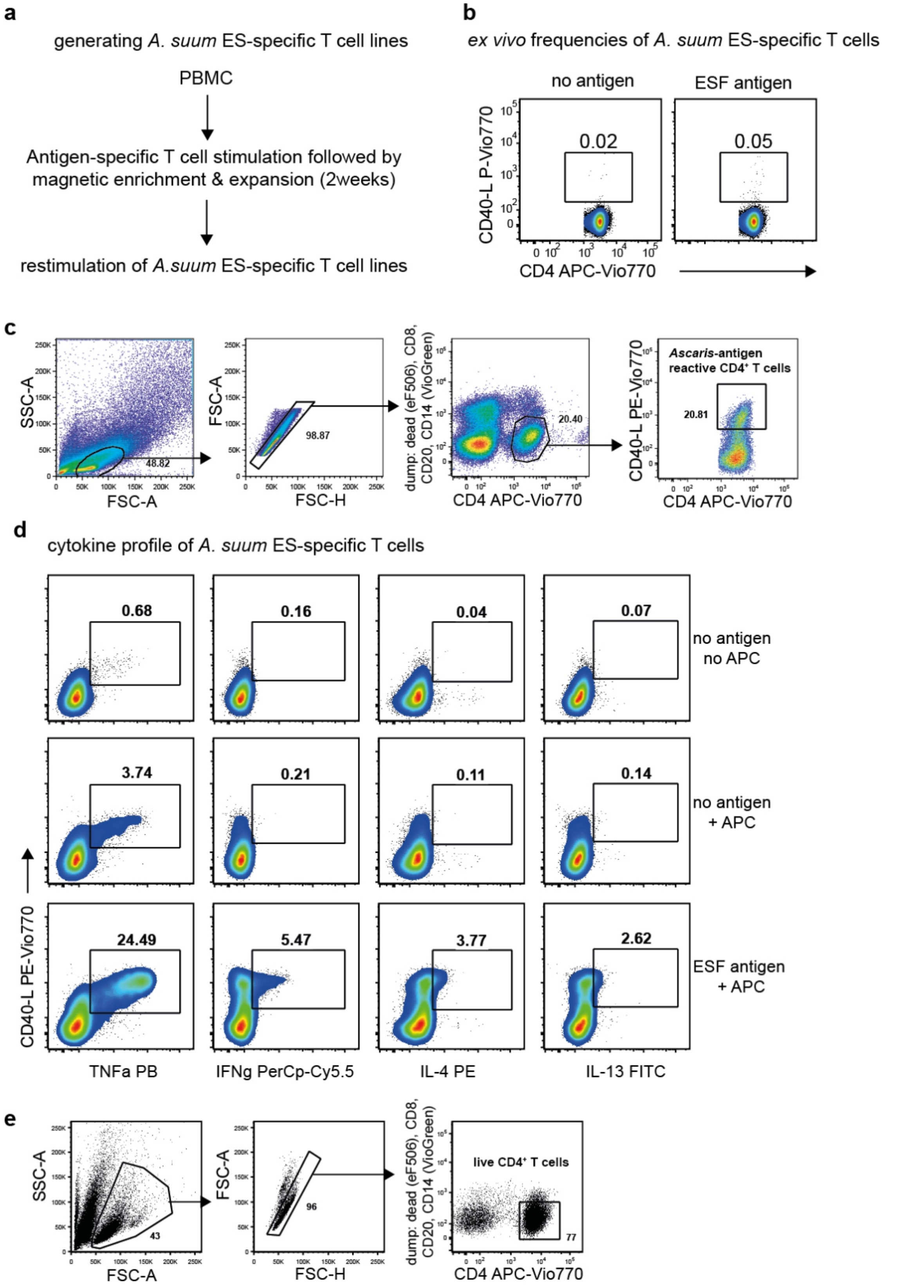

Figure 1. Additional considerations to the*Ascaris* - Antigen reactive T cell enrichment and T cell responses. a. Protocol for the generation of *Ascaris*-reactive T-cell lines. **b.** Frequencies of CD40-L expressing cells among CD4$^+$, representing *Ascaris* ES-specific T cells, were analyzed prior to expansion in unstimulated (no antigen) and *Ascaris* ESF stimulated PBMC. Numbers above gates indicate frequency of CD40L$^+$ among CD4$^+$ T cells. **c.** Gating strategy to determine percentage of CD40L$^+$ cells among CD4$^+$ T cells identifying *Ascaris*-antigen reactive T cells as analyzed in Manuscript Figures 1a, 2f, 2h (left) and Supplementary Figure 1b and 1d. **d.** *Ascaris* ESF-specific T cell lines following ESF re-stimulation gated for CD4$^+$ T cells and analyzed for CD40-L/ cytokine co-expression and compared to no antigen and no antigen/no APC controls. **e.** Gating strategy to identify live CD4$^+$ T cells prior to Tetramer-staining as presented in Figure 2h (right).

Supplementary Figure 2.

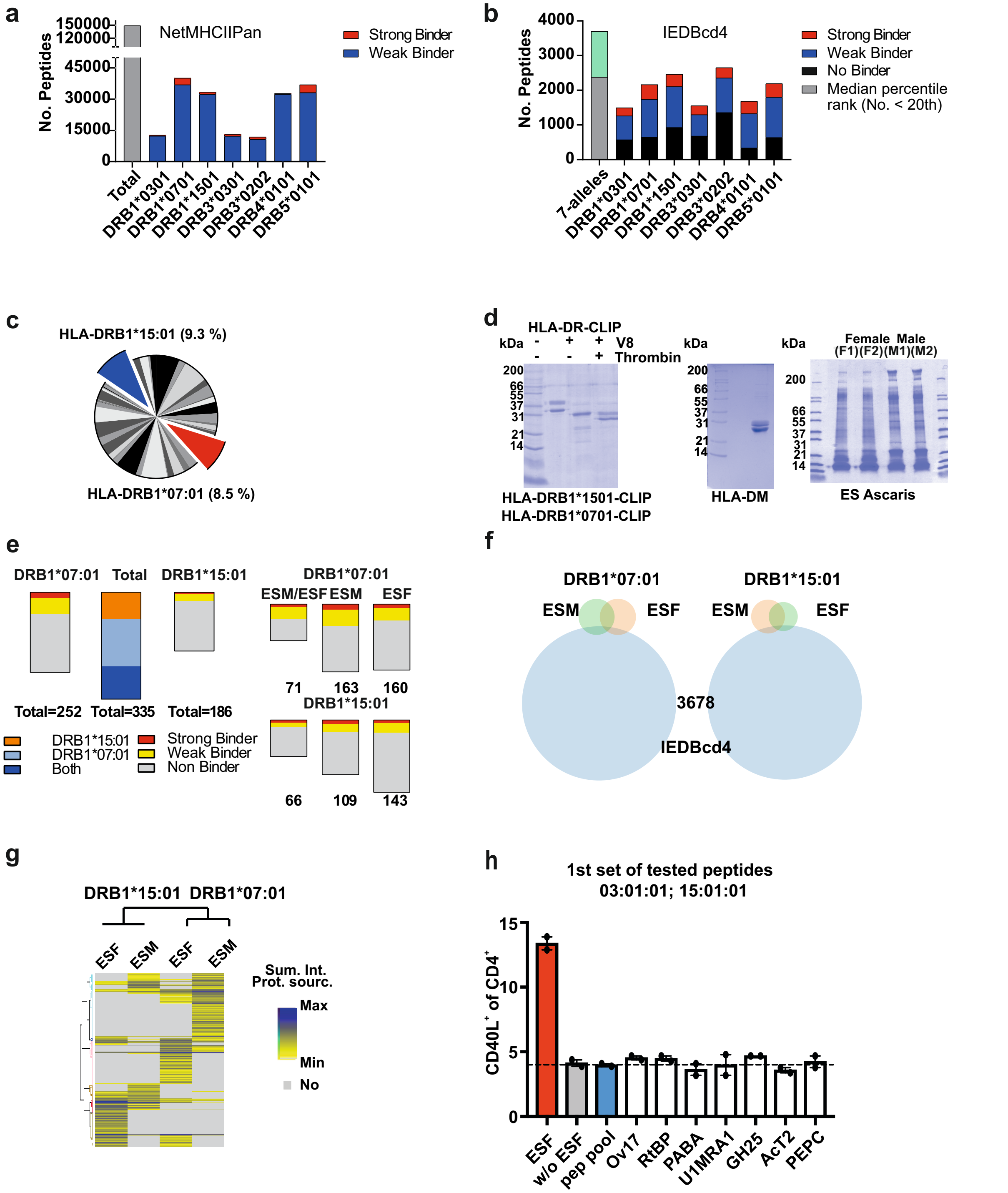

Figure 2. Additional experimental considerations for the reconstituted in vitro antigen processing system and the evaluation of its performance. a. Peptide binding affinity prediction of all entries found in the ES products of male and female worms for-7 high abundant MHCII alleles (as in ref. [27]). Total of approximately 1.5 x 10^5 15-mer peptides (gray) yield different numbers of weak (50-500 nM) and high (< 50 nM) affinity binders for each allotype (colored as shown in the legend). **b.** Prediction of Immunogenic

peptides using the IEDB CD4 T cell prediction tool (IEDBcd4) for all entries of the ES products as in (A). The relative frequency of weak and high affinity binders is shown as in (A). Additionally, the median ranked peptides for the whole allele set below the 20th percentile are shown as indicated in the figure legend and the remaining peptides are shown in green. For each allele the number of peptides included in the first 20th percentile but with a predicted affinity lower than 500 nM are shown in black (no binder). **c.** Pie chart showing the abundance of HLA-DRB1 allotypes in the 1000 Genomes Project[32] indicating the high abundance of DRB1*07:01 and DRB1*15:01 allotypes. **d.** Commassie stained SDS-PAGE showing details on the expression and purification of recombinant MHCII molecules and their processing for use on the reconstituted *in vitro* antigen processing system. DR proteins have to be cleaved with thrombin and V8 protease before being gel filtrated for their use. DM on the other hand is directly purified and gel filtrated. The left SDS-PAGE gel shows the profile of *Ascaris spp.* ES products from male and female parasites (as in Figure 1a). All gels derive from the same experiment and were processed in parallel. **e.** Detailed analysis of the epitopes found for each MHCII allotype and each antigenic source (ESF and ESM). Potential strong binders and weak binders are indicated. **f.** Full Venn diagrams shown as cropped images in Figure 2C showing the overlap between the binding cores (predicted using NetMHCIIPan) from the predicted immunogenic epitopes (IEDB immunogenicity prediction tool) and those identified experimentally. **g.** Heatmap after hierarchical clustering (Pearson correlation and Euclidean distance were applied for columns and rows respectively) of the consensus peptides selected for each condition after removing background binders. The relative abundance of each candidate is shown according to the legend. **h.** Frequency of CD40-L+ T cells responding to the 1st set of tested peptides assayed from a DRB1*15:01 donor.

Supplementary Table 1. Protein entries quantified using $^{16}O/^{18}O$ labelling.

Mascot Accession	UniProt names	Uniprot names	Student's T-test Significant Fw_Rv	Mean emPAI (min. 2)	-Log Student's T-test p-value Fw_Rv	Student's T-test Difference Fw_Rv
F1LBP8	F1LBP8_ASCSU	Chondroitin proteoglycan 3	+	0.118872212	1.67622024	-21.82127202
U1LPF4	U1LPF4_ASCSU	*Uncharacterized protein*	+	0.055008148	2.064878154	-18.62879908
U1MEA2	U1MEA2_ASCSU	*Ankyrin repeat domain-containing protein 12*	+	0.328894885	1.318808545	-16.22331798
F1L8H8	F1L8H8_ASCSU	Maleylacetoacetate isomerase 2	+	0.263743037	0.9657586	-15.24878559
U1M5G4	U1M5G4_ASCSU	*C-type lectin protein*	+	0.379334447	1.865097564	-14.99623746
F1LCS2	F1LCS2_ASCSU	Branched-chain-amino-acid aminotransferase	+	0.276957485	1.751715159	-14.92891264
F1L8V1	F1L8V1_ASCSU	Venom allergen 3	+	0.212419906	1.798632229	-13.88924241
F1KQK4	F1KQK4_ASCSU	Vitellogenin-6	+	0.355911121	1.677263725	-12.93350941
F1L144	F1L144_ASCSU	Calreticulin	+	0.478702073	1.846267225	-12.55454773
F1LEZ4	F1LEZ4_ASCSU	Peptidyl-prolyl cis-trans isomerase	+	0.653046044	1.885515421	-12.49529099
U1NQU8	U1NQU8_ASCSU	*Trypsin family protein*	+	0.378007826	2.225820843	-12.46299005
U1M491	U1M491_ASCSU	*Vitellogenin-6*	+	0.280455084	2.125237729	-12.36945739
U1LTC3	U1LTC3_ASCSU	*Uncharacterized protein*	+	0.550434042	2.047051252	-12.26203054
F1LG83	F1LG83_ASCSU	Nucleoside diphosphate kinase	+	0.355318994	1.229488438	-11.95423251
U1M8L9	U1M8L9_ASCSU	*Uncharacterized protein*	+	0.047128548	1.859659144	-11.2593981
F1L704	F1L704_ASCSU	Major sperm protein	+	0.0990242	1.806117816	-11.07902682
F1L8K5	F1L8K5_ASCSU	S-methyl-5'-thioadenosine phosphorylase	+	0.199146702	1.86072645	-11.06447309
U1N8L0	U1N8L0_ASCSU	*Phosphoenolpyruvate carboxykinase*	+	0.268664943	2.410184722	-10.95144594
Q7YXK2	Q7YXK2_ASCSU	MFP2	+	0.988816507	1.785292814	-10.89297467
F1L6F8	F1L6F8_ASCSU	Phosphotransferase	+	0.359340939	1.968962979	-10.71965468
U1NYZ1	U1NYZ1_ASCSU	*Transthyretin-like protein 16*	+	0.101309972	2.059913617	-10.50219324
F1KXK9	F1KXK9_ASCSU	Protein disulfide-isomerase	+	0.449082548	2.092105197	-10.38264585
F1KQJ9	F1KQJ9_ASCSU	Fructose-bisphosphate aldolase 1	+	0.706321532	1.690940988	-10.34660605
F1L041	F1L041_ASCSU	Phosphoenolpyruvate carboxykinase GTP	+	0.175219611	2.452008039	-10.25605774
U1M6V1	U1M6V1_ASCSU	*Uncharacterized protein*	+	0.151652312	1.802005262	-10.06062055
F1L6K0	F1L6K0_ASCSU	Pepsin inhibitor Dit33	+	0.5034771	2.07513581	-9.83156848
Q7YXJ9	Q7YXJ9_ASCSU	MFP2b	+	0.186922129	2.216079017	-9.732095271
F1LI65	F1LI65_ASCSU	Transthyretin-like protein 46	+	0.51510307	1.029458685	-9.476255816
F1LB73	F1LB73_ASCSU	Chondroitin proteoglycan 3	+	0.089022962	1.815919666	-9.28009212
F1KPM2	F1KPM2_ASCSU	Apolipophorin	+	0.811404617	1.856242838	-9.155293226
F1LEI7	F1LEI7_ASCSU	Fatty-acid and retinol-binding protein 1	+	0.873037805	2.210144888	-9.103075683
U1NWH3	U1NWH3_ASCSU	*Apolipophorin*	+	0.811542111	1.852067612	-9.07554251
W6EA75	W6EA75_ASCSU	14-3-3 zeta	+	0.770475068	1.027722515	-8.896064728
F1KXW6	F1KXW6_ASCSU	14-3-3-like protein	+	1.016126979	1.818629898	-8.82777369
F1KV03	F1KV03_ASCSU	Macrophage migration inhibitory factor	+	0.170733147	1.314019356	-8.662551671
F1L686	F1L686_ASCSU	Dihydrolipoyl dehydrogenase	+	0.382075376	2.850704561	-8.66242224
F1L5Z5	F1L5Z5_ASCSU	60S ribosomal protein L4	+	0.079591585	2.264408116	-8.634715825
F1L4K4	F1L4K4_ASCSU	C-type lectin domain-containing protein 160	+	0.511816675	1.736738466	-8.52937144
Q9NL98	PRDX_ASCSU	Peroxiredoxin	+	0.760830015	2.098155027	-7.815732241
F1KTG3	F1KTG3_ASCSU	Fibulin-1	+	0.067713231	1.880353703	-7.801382035
U1MCE3	U1MCE3_ASCSU	*ram domain containing protein*		0.014966277	0.360079625	-7.766498089
F1L2I5	F1L2I5_ASCSU	Serpin B6	+	0.748494854	1.589215389	-7.672193617
U1NMT8	U1NMT8_ASCSU	*Serpin B6*	+	0.538300409	2.017743222	-7.634984016
U1LZ59	U1LZ59_ASCSU	*Cystatin*	+	0.541804281	1.078499671	-7.575758249
F1LHQ3	F1LHQ3_ASCSU	Onchocystatin	+	0.541804281	1.010351076	-7.5559319
F1L313	F1L313_ASCSU	Neprilysin-1	+	0.584288284	1.736992054	-7.400325298
F1KVG8	F1KVG8_ASCSU	Zonadhesin		0.14212876	0.215022329	-7.342508733
F1LCY7	F1LCY7_ASCSU	Fatty acid-binding protein	+	0.337046674	1.591376117	-7.085165497
F1KY70	F1KY70_ASCSU	Alcohol dehydrogenase NADP+ A	+	0.315600349	1.797005958	-6.875112444
F1LEX9	F1LEX9_ASCSU	Transthyretin-like protein 46	+	0.834576051	1.864417354	-6.196066476
U1NWQ7	U1NWQ7_ASCSU	*Transthyretin-like protein 5*	+	0.348356564	1.44846379	-6.165120453
F1KYX0	F1KYX0_ASCSU	Acetyl-CoA acetyltransferase A		0.196294208	0.157044461	-6.105288059
F1L9M1	F1L9M1_ASCSU	Galectin	+	0.274151742	2.916523873	-6.105132073
B2REF9	B2REF9_ASCSU	Protein disulfide-isomerase	+	0.187431726	1.715659361	-6.021068215
F1KUF9	F1KUF9_ASCSU	Polyprotein ABA-1	+	1.078358904	1.459387126	-5.949634071
F1LBE9	F1LBE9_ASCSU	Heh-1	+	0.490069588	2.04921296	-5.685816467
F1LGV9	F1LGV9_ASCSU	Profilin	+	0.356287281	1.139513313	-5.551476881
F1LAR2	F1LAR2_ASCSU	OV-17 antigen		0.603680388	0.813788285	-5.36383152
F1LA15	F1LA15_ASCSU	Enoyl-CoA hydratase domain-containing protein 2		0.16875469	0.172841936	-5.266120434
U1MAD0	U1MAD0_ASCSU	*Uncharacterized protein*	+	0.33878825	1.811197957	-5.081112593
F1L8L6	F1L8L6_ASCSU	Peptidylprolyl isomerase	+	0.17394742	2.046705051	-4.762110546
F1LBT1	F1LBT1_ASCSU	H/ACA ribonucleoprotein complex subunit 4		0.046183444	0.330891357	-4.621587038
F1L246	F1L246_ASCSU	Cytosolic non-specific dipeptidase		0.185082398	0.390275787	-4.606850352
F1LEP2	F1LEP2_ASCSU	Peroxiredoxin prdx-3	+	0.184087404	1.523493857	-4.492996484
F1LFS7	F1LFS7_ASCSU	Transthyretin-like protein 5	+	0.532983154	1.255475123	-4.414939463
U1NRF9	U1NRF9_ASCSU	*Uncharacterized protein*		0.311133937	0.227088195	-4.409669042

Supplementary Table 1 (cont).

F1KRZ3	F1KRZ3_ASCSU	Huntington interacting protein related 1		0.009640276	0.111272182	-4.206784487
F1KYD8	F1KYD8_ASCSU	Protein disulfide-isomerase A6	+	0.0739337	1.912757812	-4.200822867
U1NIN4	U1NIN4_ASCSU	*Spondin-1*		0.119526633	0.161754335	-4.197883427
F1LEM2	F1LEM2_ASCSU	Metalloproteinase inhibitor tag-225		0.14926695	0.238340194	-3.491380334
F1L356	F1L356_ASCSU	Thioredoxin domain-containing protein 12		0.286724902	0.149000166	-3.473428133
F1L488	F1L488_ASCSU	Acid sphingomyelinase phosphodiesterase 3b		0.256208753	0.72624244	-3.410256635
U1MBR9	U1MBR9_ASCSU	*Nuclear hormone receptor family member nhr-121*		0.031177275	0.369518939	-3.344695061
U1MGY4	U1MGY4_ASCSU	*Uncharacterized protein*		0.027459485	0.290244703	-3.206870541
F1L7Q8	F1L7Q8_ASCSU	Histone H2B		0.092479684	0.352240864	-3.166513205
F1L5C6	F1L5C6_ASCSU	Phosphatidylethanolamine-binding protein		0.639550778	0.133744909	-3.12246573
F1LI20	F1LI20_ASCSU	Sperm-specific class P protein 19		0.215992842	0.124413936	-3.033608764
F1L963	F1L963_ASCSU	Metalloproteinase inhibitor tag-225		0.27806483	0.138493723	-2.957178339
F1KY56	F1KY56_ASCSU	Ras-related protein Rab-1A		0.126175979	0.212548977	-2.890667737
U1N9W0	U1N9W0_ASCSU	*Uncharacterized protein*		0.427480166	0.993798132	-2.694645643
F1KUF1	F1KUF1_ASCSU	Endoplasmin		0.063684354	0.127474615	-2.686757326
F1KPS3	F1KPS3_ASCSU	Cullin-1		0.010767252	0.188459525	-2.642834842
F1L0T9	F1L0T9_ASCSU	Annexin		0.120125787	0.279582852	-2.639813155
F1KSI9	F1KSI9_ASCSU	Receptor-type tyrosine-protein phosphatase F		0.055145094	0.255201826	-2.545471221
U1NG54	U1NG54_ASCSU	*Proteasome subunit alpha type*		0.195645417	0.12010626	-2.502633318
F1L518	F1L518_ASCSU	Fatty acid-binding protein		0.184937336	0.279297286	-2.459757745
F1L952	F1L952_ASCSU	Superoxide dismutase		0.232454658	0.133364619	-2.384341389
F1KPQ8	F1KPQ8_ASCSU	Mesocentin		0.144667876	1.732447066	-2.324581042
F1LB68	F1LB68_ASCSU	Alcohol dehydrogenase NADP+		0.277220753	0.157598196	-2.297177002
F1LFE6	F1LFE6_ASCSU	Transthyretin-like protein 46		0.322988292	0.135556959	-2.280224934
F1KYQ7	F1KYQ7_ASCSU	Heat shock 70 kDa protein A		0.167454713	0.233850666	-2.186968356
F1LD17	F1LD17_ASCSU	Methylmalonyl-CoA epimerase		0.13799208	0.129242453	-2.139122307
F1LIG7	F1LIG7_ASCSU	Branched-chain-amino-acid aminotransferase		0.136463666	0.090955331	-2.106439143
F1KXV2	F1KXV2_ASCSU	Triosephosphate isomerase		0.07840825	0.090546464	-1.953273058
U1NPX4	U1NPX4_ASCSU	*Aminopeptidase N*		0.539606681	0.303217691	-1.891256217
U1NNN4	U1NNN4_ASCSU	*p40*		0.196745403	0.112004173	-1.800884277
F1L7Z7	F1L7Z7_ASCSU	Proteasome subunit alpha type		0.246805992	0.493620804	-1.753657579
U1MKZ2	U1MKZ2_ASCSU	*Glutathione S-transferase 2*		0.598222757	0.645304648	-1.734172247
F1KUW6	F1KUW6_ASCSU	Glucose-6-phosphate isomerase		0.249315356	0.086079901	-1.6848104
F1LBE1	F1LBE1_ASCSU	Small heat shock protein OV25-1		0.274470095	0.116985676	-1.672144175
F1L7A0	F1L7A0_ASCSU	Superoxide dismutase [Cu-Zn]		0.929581311	1.130746292	-1.613950307
F1L3U5	F1L3U5_ASCSU	Actin-2		0.600531718	0.239774183	-1.560216472
U1P2P9	U1P2P9_ASCSU	*Lipase family protein*		0.141378419	0.056207302	-1.328500248
F1L170	F1L170_ASCSU	Nucleoporin-17		0.044402552	0.122622258	-1.274363399
F1LAD2	F1LAD2_ASCSU	32 kDa beta-galactoside-binding lectin		0.159943919	0.19090166	-1.258371151
F1L719	F1L719_ASCSU	Inorganic pyrophosphatase 1		0.605924355	0.412418784	-1.230073223
F1L2P3	F1L2P3_ASCSU	Phosphoglycerate kinase		0.14780988	0.093809083	-1.139472455
U1MDZ6	U1MDZ6_ASCSU	*Protein sidekick-1*		0.015542866	0.13320823	-1.138219148
F1LCA2	F1LCA2_ASCSU	Peroxiredoxin		0.870087537	0.056001088	-1.129771054
U1MAW1	U1MAW1_ASCSU	*Protein dj-1*		0.542258013	0.046172183	-0.952234626
U1P0K7	U1P0K7_ASCSU	*Transthyretin-like protein 16*		0.278959438	0.07056163	-0.855626028
F1KVN0	F1KVN0_ASCSU	Phosphoenolpyruvate carboxykinase GTP		0.294422614	0.042307567	-0.805486619
F1LDP8	F1LDP8_ASCSU	Actin-1		0.529324395	0.100425196	-0.761171699
F1L2P8	F1L2P8_ASCSU	Lysosomal acid phosphatase		0.160912363	0.065547001	-0.747157395
F1L0W5	F1L0W5_ASCSU	Ras-like GTP-binding protein rhoA		0.126009876	0.059988045	-0.702876121
F1L2Y8	F1L2Y8_ASCSU	Catalase		0.079239825	0.032193676	-0.648169011
F1L4M4	F1L4M4_ASCSU	Cathepsin L		0.13510944	0.034584173	-0.504365884
F1KT28	F1KT28_ASCSU	Transcription factor IIIB 90 kDa subunit		0.010644717	0.054206225	-0.491522036
F1KTS6	F1KTS6_ASCSU	TBC1 domain family member 9		0.010240508	0.059163229	-0.482479334
F1L5Q6	F1L5Q6_ASCSU	Tubulin beta chain		0.391233951	0.04940127	-0.346030816
F1L4J8	F1L4J8_ASCSU	Serpin B6		1.060326388	0.021086665	-0.289641842
U1M737	U1M737_ASCSU	*Uncharacterized protein*		0.026511068	0.049256273	-0.282604307
F1KYX4	F1KYX4_ASCSU	Cathepsin D		0.375140442	0.154611894	-0.272591889
U1MP21	U1MP21_ASCSU	*Transthyretin-like protein 15*		0.266853682	0.054840226	-0.212879956
F1KRD8	F1KRD8_ASCSU	Fumarate reductase		0.157524487	0.008897117	-0.200340033
F1L8A5	F1L8A5_ASCSU	Tubulin beta chain		0.36547312	0.028169088	-0.198468283
F1L1S5	F1L1S5_ASCSU	Serpin-like protein		1.563327986	0.068559928	-0.157870576
F1KYW3	F1KYW3_ASCSU	Sulfhydryl oxidase		0.094803486	0.041399622	-0.111675084
F1L2I1	F1L2I1_ASCSU	Alpha-galactosidase		0.167651754	0.003337641	-0.044184744
F1L7I3	F1L7I3_ASCSU	Glycine cleavage system H protein		0.205791497	0.002307619	-0.023325339
F1KTW1	F1KTW1_ASCSU	Serine protease		0.287727115	0.023025311	0.217555493
F1KWH8	F1KWH8_ASCSU	Vesicular integral-membrane protein VIP36		0.151711473	0.016836498	0.253351551
U1NPE0	U1NPE0_ASCSU	*Glutamate dehydrogenase*		0.426906584	0.068818507	0.278497893
F1LEH4	F1LEH4_ASCSU	Thyrotropin-releasing hormone-degrading ectoenzyme		0.515421103	0.05892094	0.363112003
U1LS02	U1LS02_ASCSU	Myophilin		0.225322021	0.022442041	0.376099747
F1L9H1	F1L9H1_ASCSU	Glutathione peroxidase		0.10649188	0.275520963	0.39447679
U1MCN1	U1MCN1_ASCSU	*Prostasin*		0.287626968	0.025268477	0.394985795

Supplementary Table 1 (cont).

F1L6S5	F1L6S5_ASCSU	Aminopeptidase N		0.497987051	0.137506508	0.434508048
U1M5S0	U1M5S0_ASCSU	*Fructose-bisphosphate aldolase*		1.881203628	0.254693495	0.500882238
U1MIR8	U1MIR8_ASCSU	*C-binding protein*		0.520381466	0.139345301	0.65401423
U1M1R7	U1M1R7_ASCSU	*Aminopeptidase n*		0.622622984	0.260419612	0.676196645
U1MS72	U1MS72_ASCSU	*Transaldolase*		0.215044003	0.142011056	0.761897653
U1MS59	U1MS59_ASCSU	*Uncharacterized protein*		0.113042193	0.0575906	0.77301383
F1L006	F1L006_ASCSU	Vacuolar proton pump subunit B		0.054635344	0.055757846	0.823330618
F1L0I4	F1L0I4_ASCSU	Serpin B6		0.649511399	0.306928095	0.837323457
U1NHN2	U1NHN2_ASCSU	*Maltase-intestinal*		1.688997583	0.063164189	0.873558775
F1LFA5	F1LFA5_ASCSU	Sperm-specific protein ZC168.6		0.511752747	0.268599098	0.910919428
U1N7Z6	U1N7Z6_ASCSU	*Uncharacterized protein*		0.1440726	0.099707657	0.988738179
U1MUX9	U1MUX9_ASCSU	*Uncharacterized protein*		1.242462887	0.153530921	1.062660456
F1LBG3	F1LBG3_ASCSU	OV-16 antigen		0.373542155	0.563631459	1.167961486
U1NGG6	U1NGG6_ASCSU	*Uncharacterized protein*		0.072123454	0.061182227	1.252186172
F1KPJ7	F1KPJ7_ASCSU	Titin		0.027632405	0.076132333	1.268729329
F1L602	F1L602_ASCSU	Tubulin beta chain		0.147038159	0.632105298	1.278757572
U1M8X5	U1M8X5_ASCSU	*Acid phosphatase*		0.23176309	0.161945269	1.443450153
F1L4H4	F1L4H4_ASCSU	ATP synthase subunit beta		0.122605938	1.018560263	1.455876261
F1KZ94	F1KZ94_ASCSU	ADP/ATP translocase 2		0.02305973	1.319606847	1.498760223
F1LEB4	F1LEB4_ASCSU	Transthyretin-like protein 16		0.186569162	0.671894956	1.550278544
F1L5N0	F1L5N0_ASCSU	Tubulin alpha chain		0.13475613	1.636991801	1.723395884
F1L4K1	F1L4K1_ASCSU	Histone H4		0.333521432	1.213565204	1.771351814
U1LYA8	U1LYA8_ASCSU	*Immunoglobulin i-set domain containing protein*		0.14481264	0.195368927	1.779658377
F1L7E0	F1L7E0_ASCSU	Annexin		0.173728459	1.172508907	1.788817644
F1L5P4	F1L5P4_ASCSU	SUMO-conjugating enzyme UBC9		0.066050499	0.643166893	1.83306551
F1KTQ3	F1KTQ3_ASCSU	Maltase-glucoamylase-like protein		0.184546345	0.700788682	1.869837731
F1LEJ6	F1LEJ6_ASCSU	Polyubiquitin		0.244164469	0.656028017	1.869900838
U1NL82	U1NL82_ASCSU	*Aminopeptidase n*		0.926051328	1.851120372	2.042116433
F1KQN0	F1KQN0_ASCSU	CD109 antigen		0.122218759	0.785603019	2.104005158
U1NJP5	U1NJP5_ASCSU	*Uncharacterized protein*		0.236226077	0.170011239	2.148610849
F1LHC8	F1LHC8_ASCSU	Transthyretin-like protein 15		1.025675131	1.870748898	2.263593897
F1L5G6	F1L5G6_ASCSU	Tubulin alpha chain		0.170828137	3.1166632	2.282004029
F1LFI4	F1LFI4_ASCSU	Transthyretin-like protein 5		0.201532248	0.889595522	2.2943617
F1L040	F1L040_ASCSU	Serpin-like protein		0.52027145	0.638907641	2.301630966
F1L4U1	F1L4U1_ASCSU	Lipase		0.190143106	0.23478745	2.301708676
F1LHW8	F1LHW8_ASCSU	Transthyretin-like protein 16		0.656800706	1.003402735	2.505689234
F1L893	F1L893_ASCSU	32 kDa beta-galactoside-binding lectin		0.24922586	2.65417029	2.544585004
U1LWU6	U1LWU6_ASCSU	*Uncharacterized protein*		0.21180302	2.448538163	2.601306319
F1KX42	F1KX42_ASCSU	Moesin/ezrin/radixin 1		0.056309846	0.507564265	2.615634292
F1KTY6	F1KTY6_ASCSU	Maltase-glucoamylase		0.196804924	0.652410467	2.625859722
F1L6P7	F1L6P7_ASCSU	Elongation factor 1-alpha		0.088292654	1.181137778	2.709197596
U1MRA1	U1MRA1_ASCSU	Uncharacterized protein		1.563505852	1.047350674	2.727307916
F1L5X8	F1L5X8_ASCSU	Ribonuclease UK114		0.369383068	0.747539434	2.770823598
F1L0N2	F1L0N2_ASCSU	Serpin-like protein		1.236766421	0.625679843	2.811110139
U1M6U4	U1M6U4_ASCSU	*Myophilin*		0.405340015	1.286296491	2.961176336
F1KU32	F1KU32_ASCSU	C-type lectin domain-containing protein 160		1.375784177	0.661878315	3.01627674
U1M2W9	U1M2W9_ASCSU	*Putative serpin-like protein ma 3388*		1.211509555	0.626246608	3.093382426
U1MF79	U1MF79_ASCSU	*Aminopeptidase n*		0.32514803	0.908898486	3.096341662
F1KQL5	F1KQL5_ASCSU	Aminopeptidase N		1.473945077	1.198371406	3.37699163
F1KUR2	F1KUR2_ASCSU	Zonadhesin		0.426500042	1.026284482	3.459985971
F1KR19	F1KR19_ASCSU	Thyrotropin-releasing hormone-degrading ectoenzyme		1.124430652	1.096050654	3.462932318
F1KPH3	F1KPH3_ASCSU	Mesocentin		0.041401923	0.478449257	3.490403995
F1L2P6	F1L2P6_ASCSU	Carboxypeptidase		0.512910805	1.07419611	3.547281265
U1MMA2	U1MMA2_ASCSU	*Uncharacterized protein*		0.253894087	0.215772886	3.777059972
F1L0R7	F1L0R7_ASCSU	C-type lectin protein 160		0.870137874	1.183284873	3.789081454
F1LGV6	F1LGV6_ASCSU	Histone H2A		0.068000433	1.129049067	3.817809284
U1MI86	U1MI86_ASCSU	*Uncharacterized protein*	+	0.557473164	2.192230294	3.847957663
F1KQD3	F1KQD3_ASCSU	Aminopeptidase N	+	0.413761271	1.290814851	3.884162292
F1L7K1	F1L7K1_ASCSU	C-type lectin protein 160	+	0.313730833	4.430232849	3.911290914
U1MJ81	U1MJ81_ASCSU	*Prolyl carboxy peptidase like protein*	+	0.257198696	2.001748641	4.003259867
F1L837	F1L837_ASCSU	Carbonic anhydrase 5		0.745271673	0.908248924	4.006358653
U1LZY0	U1LZY0_ASCSU	*Uncharacterized protein*		0.060818355	0.865563443	4.038188577
U1P3X1	U1P3X1_ASCSU	*Uncharacterized protein*	+	0.767300289	1.323663494	4.19318294
F1LE63	F1LE63_ASCSU	GH family 25 lysozyme 2	+	1.300657441	1.120513819	4.225597836
U1M5W7	U1M5W7_ASCSU	*Uncharacterized protein*	+	0.394787808	1.715316253	4.242057575
F1L1P8	F1L1P8_ASCSU	Serine protease	+	0.402631958	1.629233223	4.246434063
F1L4Z6	F1L4Z6_ASCSU	Trehalase	+	0.311300982	2.287828431	4.334164351
U1P2Q0	U1P2Q0_ASCSU	*Cre-clc-1 protein*		1.227689819	0.950208804	4.382950753
F1LCX2	F1LCX2_ASCSU	Glutathione S-transferase 1		1.722571867	1.041454767	4.41476357
F1L023	F1L023_ASCSU	C-type lectin protein 160	+	0.179998078	1.534283371	4.43616315
U1M7B8	U1M7B8_ASCSU	*Glutathione s-transferase 1*		1.95684579	1.029187889	4.449808866

Supplementary Table 1 (cont).

F1L697	F1L697_ASCSU	C-type lectin protein 160	+	0.430794898	1.111309812	4.601505741
U1M2X2	U1M2X2_ASCSU	*Alpha-glucosidase*	+	0.65469781	1.610834294	4.710990161
F1KST6	F1KST6_ASCSU	ATP-dependent DNA helicase	+	0.01163798	1.321561484	4.761460841
U1M7Y4	U1M7Y4_ASCSU	*Annexin*	+	0.11589695	2.68110904	4.803602099
U1MBM9	U1MBM9_ASCSU	*Superoxide dismutase [Cu-Zn]*		0.250093217	0.864119535	4.848685145
U1M5T8	U1M5T8_ASCSU	*C-type lectin protein*	+	0.382380012	1.200798472	4.854945526
F1KQT8	F1KQT8_ASCSU	Neprilysin-1	+	0.894392866	1.884296792	4.968792468
F1L2X0	F1L2X0_ASCSU	Maltase-glucoamylase	+	0.420315196	1.326058383	5.172883868
F1L7L2	F1L7L2_ASCSU	Annexin	+	0.291007708	3.803659469	5.248697579
F1KPY0	F1KPY0_ASCSU	Carboxypeptidase	+	0.547416426	1.181363938	5.248851061
U1NJ58	U1NJ58_ASCSU	*Serpin b6*	+	1.032966779	1.132519355	5.281097174
F1L7R9	F1L7R9_ASCSU	C-type lectin domain-containing protein 160	+	0.447160866	1.907432931	5.308440946
U1P3P5	U1P3P5_ASCSU	*Maltase-intestinal*	+	1.515341349	1.262665778	5.52313301
F1KPL9	F1KPL9_ASCSU	Neprilysin-1	+	0.655328072	1.52080875	5.541521907
F1KPW3	F1KPW3_ASCSU	Maltase-glucoamylase	+	1.504707238	1.711367918	5.61144039
F1KRB6	F1KRB6_ASCSU	Sucrase-isomaltase	+	1.538627726	1.853846256	5.622577041
F1L6X4	F1L6X4_ASCSU	Serpin-like protein	+	1.325233267	1.057702242	5.650586247
U1NF90	U1NF90_ASCSU	*Aspartic protease*	+	0.462148922	1.131847285	5.704854071
F1KX94	F1KX94_ASCSU	Sucrase-isomaltase	+	1.097614059	1.859618018	5.741840124
F1L2M0	F1L2M0_ASCSU	Serine protease	+	0.367788469	1.215234299	5.952421486
F1KUA0	F1KUA0_ASCSU	Sucrase-isomaltase	+	1.906085995	1.157141652	6.188556924
U1M8Q9	U1M8Q9_ASCSU	*Serpin b3*	+	0.395055721	1.124499976	6.382649377
P28316	GLB_ASCSU	Extracellular globin	+	0.53970261	1.062812896	6.469806433
U1M8R0	U1M8R0_ASCSU	*Sucrase-intestinal*	+	1.860972126	1.259254424	6.486659177
F1L771	F1L771_ASCSU	Extracellular globin		0.345405598	0.672583324	6.780021325
U1MBW8	U1MBW8_ASCSU	*Uncharacterized protein*		0.267473138	0.375851495	6.994741559
F1KSB8	F1KSB8_ASCSU	AP complex subunit beta	+	0.047314805	1.940226118	7.053487778
F1LBJ8	F1LBJ8_ASCSU	Cytochrome c type-1	+	0.479677823	2.580061124	7.234355509
F1KVU5	F1KVU5_ASCSU	Aspartic protease 6	+	0.790077754	1.188022742	7.604774922
F1LF53	F1LF53_ASCSU	Nucleoredoxin-like protein 2	+	0.489668779	0.774501445	8.11801821
F1KQE6	F1KQE6_ASCSU	Nidogen-1	+	0.04415934	1.690572517	8.773495913
F1L723	F1L723_ASCSU	Aspartic protease 6	+	0.645848096	1.062532707	8.968650758
U1MA91	U1MA91_ASCSU	*Vacuolar protein sorting-associated protein 52-like protein*	+	0.0797429	1.868237638	9.358686566
F1L3M2	F1L3M2_ASCSU	Proteasome subunit alpha type		0.204564728	0.479414256	9.375122428
U1MJD0	U1MJD0_ASCSU	*Uncharacterized protein*	+	0.634235473	1.180364057	9.429013014
F1L1C2	F1L1C2_ASCSU	Poly(U)-specific endoribonuclease	+	0.523160182	2.474388017	9.5603019
F1KYK1	F1KYK1_ASCSU	Polycomb group RING finger protein 2	+	0.019705064	1.063552765	10.88429582
F1KPF4	F1KPF4_ASCSU	Basement membrane proteoglycan	+	0.117502783	2.779040407	12.67333865
F1KR62	F1KR62_ASCSU	Collagen alpha-1(XVIII) chain	+	0.086955408	2.69796351	14.18316662
F1LA05	F1LA05_ASCSU	Disorganized muscle protein 1	+	0.505209466	2.070204543	14.41758776
F1LCY4	F1LCY4_ASCSU	Glutathione S-transferase 4	+	0.192989448	2.232443712	14.55558848
F1KPE9	F1KPE9_ASCSU	Titin	+	0.075643565	1.872005808	14.61745894
F1LDH6	F1LDH6_ASCSU	Ancylostoma secreted protein	+	0.911552396	1.813156186	15.06520939
F1L7M6	F1L7M6_ASCSU	Cuticle collagen 12	+	0.245197085	1.710271353	15.17406321
F1LDV0	F1LDV0_ASCSU	Cuticle collagen 34	+	0.36887451	1.757491163	16.43138206
Q9U994	Q9U994_ASCSU	Putative cuticular collagen	+	0.324192792	1.422003393	23.11077714

Protein entry names are indicated in the first column and their common names on the third one (italized names are entries that have been modified in the course of the research presented here). Significantly enriched proteins are indicated with a "+" in the corresponding column (enriched). Values for the statistcs and calculated emPAI are also indicated as well as the emPAI (minimum count for calculation is 2). ESF enriched proteins have negative values and ESM enriched are shown with positive values.

Supplementary Table 2. Consensus peptides defined by PLAtEAU after filtering background binders and controls

Consensus peptide	Proteins	Lead Protein	Lead Protein Start Pos.	Lead Protein End Pos.	Core epitope length	DR7_ESM	DR7_ESF	DR15_ESM	DR15_ESF	Source enriched	Source Abundance	Pred_Aff_DR7	Pred_Aff_DR15	Predicted_DR7_Core	Predicted_DR15_Core	IEDB_I_DR7	IEBD_I_DRB15	Peptide tested
YFDHDNAKDPLFASVSNRINDGPTF	F1L1C2	F1L1C2	69	83	25	0.0267735	0.0001151	6.15E-05	0.0003	Male	Medium	<=WB		LFASVSNRI	LFASVSNRI	+		
YDTHSLYGTWMAIQTQKAQEE	F1KUA0;U1M8R0	F1KUA0	346	357	21	0.0185608	0.0046	0.00025	0.00017	Male	High			WMAIQTQKA	WMAIQTQKA	+		
**SYFVKSTRTKVLLADDENPAEQ**	**U1MRA1**	**U1MRA1**	**611**	**618**	**23**	**0.0383561**	**0.0020489**	**0.00135**	**0.00011**	**No**	**High**	**<=WB**		**VKSTRTKVL**	**VKSTRTKVL**	**+**		**SYFVKSTRTKVLLLA**
AASNDLFQQTIVKVISAKTQVVAG	F1LHQ3;U1LZ59	F1LHQ3	50	65	24	0.00074863	0.0012837	0.00057	0.00113	Female	Medium	<=WB	<=SB	IVKVISAKT	IVKVISAKT	+		
FKGPNGYISPWHDIPLFADEAKK	F1L719;Q86M43	F1L719	125	138	23	0.00047326	0.0006002	0.00127	0.018	No	Medium			ISPWHDIPL	ISPWHDIPL	+		
DSQKSELESMRAAGSSFDEIHEK	F1KTI9;F1KUF9	F1KTI9	345	367	23	0.00014835	0.0088469	0.00031	0.01326	Female	High			LESMRAAGS	LESMRAAGS	+		
ATIEKVRALGANIGM	F1LE63;F1LEA7	F1LE63	95	106	15	0.0638204	0.139653	0.00514	0.00612	Male	High	<=WB	<=WB	IEKVRALGA	VRALGANIG	+		
WMDIERFAWPSNK	F1LE63;F1LEA7	F1LE63	95	106	13	0.029779	0.0410127	0.00459	0.00151	Male	High			IERFAWPSN	IERFAWPSN	+		
NQVQATIEKVRALGANIG	F1LE63;F1LEA7	F1LE63	95	106	18	0.0395495	0.104531	0.00077	0.0022	Male	High			IEKVRALGA	IEKVRALGA	+		
**MTEQQKQQIRSMESSNVAPLEIKKK**	**F1KTI9;F1KUF9**	**F1KTI9**	**90**	**102**	**25**	**0.023425**	**0.0807366**	**0.00522**	**0.48027**	**Female**	**High**	**<=WB**	**<=WB**	**IRSMESSNV**	**IRSMESSNV**	**+**		**KQQIRSMESSNVAPL**
WFQGDGMVRRKNLPIEYNL	F1LGA1;F1LIE3;F1LIE5;P27439;P27440;Q53ZF4;U1N6W5	F1LGA1	120	138	19	-	0.021368	-	0.04166					MVRRKNLPI	MVRRKNLPI			
FVEATGNSPLEITRSAGPPKND	Q6BD87;Q6BD89;Q6BD92;Q6BD94;Q6BD96;Q8ISC1	Q6BD87	102	123	22	7.40E-06	0.0121505	1.06E-06	0.00107					VEATGNSPL	ITRSAGPPK	+		
VGSALSDPYLSFSASMAGLAGPLH	U1MH21	U1MH21	305	328	24	7.06E-05	0.0047417	-	0.00191			<=SB	<=WB	FSASMAGLA	YLSFSASMA	+		
ARDLTTDEIIELKEFDAKMSEYQK	F1L6K0	F1L6K0	154	165	24	0.00037653	0.0237007	9.80E-06	0.02265	Female	Medium			LKEFDAKMS	LKEFDAKMS	+		
SIGENHAQYSSIDAALDALKE	F1LEI7	F1LEI7	58	78	21	3.43E-05	0.0142643	-	0.01807	Female	Medium			YSSIDAALD	YSSIDAALD	+		
**LEAKILHYYDELEGDAKKE**	**F1KTI9;F1KUF9;Q06811**	**F1KTI9**	**934**	**952**	**19**	**9.81E-06**	**0.0008341**	**0.55626**	**2**	**Female**	**High**			**LHYYDELEG**	**ILHYYDELE**	**+**		**AKILHYYDELEGDAKKEATEH**
DKQILRSIGENHAQY	F1LEI7	F1LEI7	52	66	15	-	0.0002687	0.00173	0.21043	Female	Medium			ILRSIGENH	LRSIGENHA			
MGPGGSIARIVSPQDYPYVVE	U1NQU8	U1NQU8	64	82	21	-	0.00096	0.01475	0.03527	Female	Medium			IARIVSPQD	IARIVSPQD			
TAVREVIEKYKTLPEATKQE	F1LEI7	F1LEI7	134	153	20	-	2.77E-05	0.00015	1.04857	Female	Medium			IEKYKTLPE	IEKYKTLPE			
LIGQIVSSITASLR	F1L5N0;F1L868;U1NXZ9	F1L5N0	256	269	14	1.61E-05	4.59E-05	0.00063	0.001	No	Medium	<=SB	<=WB	IVSSITASL	IVSSITASL	+	+	
DKSISSQTTFIQSHLLKQ	U1MRA1	U1MRA1	534	551	18	1.97E-05	2.77E-05	0.00015	0.00078	No	High			TTFIQSHLL	TTFIQSHLL	+		
MMSLDSLTFGQWRKTTAQIGF	F1KQK4;F1KQP6;U1MGQ2	F1KQK4	746	766	21	-	0.0010798	0.00081	0.00101	Female	Medium			WRKTTAQIG	WRKTTAQIG			
VELLRWQGEDISPDRDGT	F1L8L6;U1N6X9	F1L8L6	116	133	18	1.68E-06	3.04E-05	0.0004	0.00278	Female	Medium			LLRWQGEDI	LLRWQGEDI	+		
SNIDWLSKNRDEIIDWINQNLN	F1KQF7;U1NL82	F1KQF7	1750	1771	22	-	0.0001579	0.00121	0.00321	No	Medium			LSKNRDEII	IIDWINQNL			
GRDITFSSFYETPGQTLYWKMPTPF	F1KPF4;F1KPG3;F1KPK5	F1KPF4	911	926	25	0.00016804	-	0.00068	0.00045	Male	Medium			FYETPGQTL	FYETPGQTL			
**VAPITSFMTPLDTYQLAPT**	**U1MRA1**	**U1MRA1**	**611**	**618**	**19**	**9.21E-06**	**-**	**0.0008**	**0.0002**	**No**	**High**	**<=WB**		**MTPLDTYQL**	**ITSFMTPLD**			**PITSFMTPLDTYQLAPTLQLL**
GDVLQYKDSDFEDSIKG	F1KXK9	F1KXK9	17	33	17	-	-	0.00068	0.00836	Female	Medium			YKDSDFEDS	VLQYKDSDF			
LAVNMVPFPR	F1L4P8;F1L5Q6;F1L5R2;F1L602;F1L7U3;F1L8A5;F1LDP6;F1LDW0;F1LH54;F1LHP4;U1N9C8	F1L4P8	310	319	10	-	-	0.00153	0.00401	No	Medium			AVNMVPFPR	AVNMVPFPR			
DVNAAIATIK	F1L5N0;F1L868;F1LH98;U1NXZ9	F1L5N0	353	362	10	-	-	0.00143	0.00253	No	Medium			VNAAIATIK	VNAAIATIK			
LRQQAVAIKGKLLCGTAPA	F1LEX9;U1P0G4	F1LEX9	26	44	19	-	-	0.00223	0.00178	Female	Medium			AVAIKGKLL	AVAIKGKLL			
AVCMLANTTAIAEAWAR	F1L5G6;F1LHF5	F1L5G6	443	459	17	-	-	0.0002	0.00029	No	Medium			CMLANTTAI	MLANTTAIA			
TVLIMELINNVAK	F1L4H4	F1L4H4	234	246	13	-	-	0.00032	0.00037	No	Medium		<=WB	LIMELINNV	IMELINNVA			
AITVPELTQQMFDAK	F1L5Q6	F1L5Q6	211	225	15	-	-	0.00027	0.00096	No	Medium			TVPELTQQM	TVPELTQQM			
DNIQGITKPAIR	F1L4K1;F1L914;F1LC49;Q27443	F1L4K1	25	36	12	-	-	0.0002	0.00074	No	Medium			IQGITKPAI	IQGITKPAI			
LTPHKNYFVYEYASHVLPRPYK	F1L0R7	F1L0R7	430	445	22	-	-	0.00083	0.00054	No	Medium	<=SB	<=SB	FVYEYASHV	FVYEYASHV		+	
RYFYNQEESVRFDSDVGEFRAVT	DRB*01:15	DRB*01:15	83	105	23	-	-	0.0117	0.47079					YNQEESVRF	YNQEESVRF			
SDVGEFRAVTE	DRB*01:15	DRB*01:15	96	106	11	-	-	0.02828	0.57043					VGEFRAVTE	VGEFRAVTE			
EDEVGSIEPY	F1LHC8	F1LHC8	91	100	10	-	-	0.99155	0.03922	No	High			DEVGSIEPY	DEVGSIEPY			
VFVIDVSRAVSLQDF	U1M5G4	U1M5G4	336	348	15	-	0.0001739	-	2.55E-05	Female	Medium	<=SB		IDVSRAVSL	FVIDVSRAV		+	
ARIFGSDGLLWNG	U1NRF9	U1NRF9	79	91	13	-	2.95E-05	-	8.45E-05	No	Medium			IFGSDGLLW	IFGSDGLLW			
ASVLKLVVRSLSQVVQSGAQN	F1LAK0	F1LAK0	186	206	21	-	4.51E-05	-	6.25E-05					VVRSLSQVV	VVRSLSQVV			
AESAVFRSWNADIVNM	F1L8K5;U1NK84	F1L8K5	203	218	16	-	5.26E-06	-	5.30E-05	Female	Medium		<=WB	FRSWNADIV	FRSWNADIV		+	
**YLYKGQISNGL**	**F1KQS3;U1M491**	**F1KQS3**	**41**	**51**	**11**	**-**	**1.79E-05**	**-**	**4.32E-05**	**Female**	**Medium**	**<=WB**		**YKGQISNGL**	**YKGQISNGL**			**RYLYKGQISNGLSKL**
TADGPILFDYSKNLIDE	F1KUW6;U1NWT5	F1KUW6	68	84	17	-	9.81E-06	-	6.58E-05	No	Medium			FDYSKNLID	ILFDYSKNL			
EAAADAPDATVIFKEGAPLGE	F1LI20;Q7YXK3	F1LI20	79	99	21	-	2.82E-05	-	0.00114	No	Medium			VIFKEGAPL	VIFKEGAPL			
SSSLGKFDYQIFSKLEENE	F1KPM2	F1KPM2	1176	1194	19	-	5.84E-05	9.58E-07	0.0002	Female	Medium			FDYQIFSKL	FDYQIFSKL			
VSGFPTLKIFRNGVFAQDYDGPRE	F1KXK9	F1KXK9	89	112	24	-	4.41E-05	-	0.00019	Female	Medium		<=WB	LKIFRNGVF	LKIFRNGVF			
KIFEKPIIFKTLVNLAAPQYQ	F1KPM2	F1KPM2	1087	1107	21	-	5.31E-05	-	0.00027	Female	Medium		<=WB	FKTLVNLAA	FKTLVNLAA		+	
ASSGESLSDYITSHPSSLGAHEDG	F1L859	F1L859	59	82	24	-	2.76E-05	-	0.00017					YITSHPSSL	ITSHPSSLG			
LKQIATAILASGKGILAADESNPS	F1KQJ9;U1MCE0	F1KQJ9	17	40	24	-	2.08E-05	-	0.00037	Female	Medium			LASGKGILA	ILASGKGIL			
LNNDRITIEWTNTPDGAAK	F1LEI1;F1LGA1;F1LIE3;F1LIE5;P27439;P27440;Q53ZF4;U1N6W5	F1LEI1	85	103	19	-	9.46E-05	-	0.00041					IEWTNTPDG	ITIEWTNTP			
VGAGVNAFDARNIITK	U1MEA2	U1MEA2	60	75	16	-	0.0006154	-	0.00039	Female	Medium			VNAFDARNI	VNAFDARNI			
QQALEAAKSAAVGSVPQ	Q93122	Q93122	76	92	17	7.87E-06	0.0009295	-	0.00024					LEAAKSAAV	LEAAKSAAV	+		
**MNSEDKSQILDYKVTSASGIFEVE**	**U1LVE6**	**U1LVE6**	**47**	**68**	**24**	**4.13E-06**	**0.0007683**	**2.19E-06**	**0.00045**			**<=SB**		**YKVTSASGI**	**YKVTSASGI**	**+**		**ILDYKVTSASGIFEV**
HYLKTYLSWLTEEQKEKLKE	F1KTI9;F1KUF9;Q06811	F1KTI9	634	653	20	-	0.0001426	-	0.0025	Female	High			LKTYLSWLT	LKTYLSWLT			
AQIINTLPQRAVLDQ	F1KQS3;U1M491	F1KQS3	637	651	15	-	2.73E-05	-	0.01541	Female	Medium			IINTLPQRA	IINTLPQRA		+	
VGNIEPYLLISH	F1LHW8;U1MA99	F1LHW8	60	71	12	-	2.88E-05	-	0.00335	No	Medium			IEPYLLISH	IEPYLLISH			
ILKHVVGEEKAAELKNLKDSGASKEEL	F1KTI9;F1KUF9;Q06811	F1KTI9	964	990	27	-	5.81E-05	-	0.00606	Female	High			LKDSGASKE	LKNLKDSGA			
AQVIHTLPERTILDQ	F1KQK4;F1KQP6;U1MGQ2	F1KQK4	641	655	15	-	6.64E-05	-	0.00805	Female	Medium			VIHTLPERT	VIHTLPERT			
AQALLEWIKKLSGENISTSGDREN	U1M6U4	U1M6U4	27	50	24	-	-	0.00016	0.00012	No	Medium			IKKLSGENI	IKKLSGENI			
TIQNFPAFRTA	F1KPL9	F1KPL9	1435	1445	11	-	-	7.83E-05	4.76E-05	Male	Medium	<=WB	<=SB	IQNFPAFRT	IQNFPAFRT			
YVQVFGASNVFDGSAIKSGPVTA	U1MRA1	U1MRA1	676	698	23	-	-	8.79E-05	5.90E-05	No	High			VQVFGASNV	VQVFGASNV		+	
PNAIANQIKE	F1KU32;F1L8I9;F1LGY6;U1NRL6	F1KU32	131	140	10	-	-	7.61E-06	3.87E-05	No	High			NAIANQIKE	NAIANQIKE			
ANGLAALGKYTGGGAGSAAG	F1L5H2;F1LCU9;U1M5S0	F1L5H2	348	359	20	-	-	6.97E-06	7.51E-05	No	High			YTGGGAGSA	LGKYTGGGA			
FVDNVFPHHGYIWNYGALPQTWED	F1L719;F1LH74;Q86M43	F1L719	179	202	24	8.47E-06	-	3.42E-05	0.00023	No	Medium			YIWNYGALP	YIWNYGALP			
IVFASSQGENYANAVTE	U1MRA1	U1MRA1	158	166	17	-	1.70E-05	2.20E-05	1.02E-05	No	High			FASSQGENY	FASSQGENY			
TFQSVDLFEGRDLFS	U1M6U4	U1M6U4	106	120	15	-	-	9.70E-06	0.00429	No	Medium			VDLFEGRDL	VDLFEGRDL			
LDIMQILDPHALDK	F1L8V4;U1MKZ2	F1L8V4	118	131	14	-	-	1.95E-06	0.00623	No	Medium			MQILDPHAL	IMQILDPHA			
KKDEEKTDPEIEADIDAFVAKLGAD	F1LAR2;Q86M44	F1LAR2	90	114	25	-	-	-	0.00585	No	Medium			IDAFVAKLG	IDAFVAKLG			
YKVGQYVENYFSRLLDMLKA	F1KQK4;U1MGQ2	F1KQK4	1369	1388	20	-	-	-	0.00269	Female	Medium			YVENYFSRL	VENYFSRLL			
DAALDALKEKSEKLHAK	F1LEI7	F1LEI7	70	86	17	-	-	-	0.00276	Female	Medium			ALKEKSEKL	ALKEKSEKL			
AEVASGDDRNVVVEKSQQSYQE	F1KXW6;F1LAU0;F1LF96;W6EA75	F1KXW6	137	156	22	-	-	-	0.00338	Female	High			VVVEKSQQS	VVVEKSQQS			
YELPEAIRGFDYDWKPFPE	Q7YXJ9	Q7YXJ9	273	291	19	-	-	-	0.01542	Female	Medium			PEAIRGFDY	IRGFDYDWK			
NEIEAMLSGQLKDLQT	F1LB68	F1LB68	90	105	16	-	-	-	0.02052	No	Medium			IEAMLSGQL	IEAMLSGQL			
YDSDVTVWSPQGRLHQ	F1L3M2;U1NJ88	F1L3M2	6	21	16	-	-	1.37E-06	0.00011	No	Medium			TVWSPQGRL	VTVWSPQGR			
IGPKFTAYDSADAE	U1MIR8	U1MIR8	672	685	14	-	-	-	5.66E-05	No	Medium			FTAYDSADA	FTAYDSADA			
EVRPALMNYFNNIF	U1M491	U1M491	1329	1342	14	-	-	-	6.30E-05	Female	Medium		<=WB	VRPALMNYF	LMNYFNNIF		+	
IDTAYVYGNEAVIGK	F1LB68	F1LB68	46	60	15	-	-	-	6.43E-05	No	Medium			YVYGNEAVI	AYVYGNEAV			
NQDEMRDWIMPVGFDH	F1L5H3	F1L5H3	245	260	16	-	-	-	5.76E-05					WIMPVGFDH	WIMPVGFDH			
IPIISDNNHQISRD	F1L801;F1LCA2;F1LDA3;F1LFB4;F1LGD2;F1LI98;Q9NL98	F1L801	99	112	14	-	-	-	5.15E-05	No	Medium			IISDNNHQI	IISDNNHQI		+	
ATVIFKEGAPLGEVVLPLN	F1LI20;Q7YXK3	F1LI20	87	105	19	-	-	-	7.49E-05	No	Medium			FKEGAPLGE	VIFKEGAPL			
GDINTQPSQKIVFNAPYDDK	F1LEI1;F1LHB2;P27439;Q53ZF4	F1LEI1	8	27	20	-	-	-	8.28E-05					INTQPSQKI	INTQPSQKI			
SVSNSEKDIMAEIYKNGPVEGAFSV	P07858	P07858	231	255	25	-	-	-	8.30E-05					IYKNGPVEG	IYKNGPVEG			
GIVKYMRGQAGPSA	F1KXK9	F1KXK9	115	128	14	-	-	-	8.26E-05	Female	Medium		<=WB	VKYMRGQAG	VKYMRGQAG		+	
AEREIWSFGSGYGGNSL	F1KSI5;F1KVN0;F1KXJ0;F1L041;F1L1Z3;F1LEQ5;Q05893;U1MDY4;U1N8L0;U1NR83	F1KSI5	241	257	17	-	-	-	8.19E-05	Female	Medium			WSFGSGYGG	IWSFGSGYG			
YVRRGDLDYAVLPMDAETMPEL	F1KQK4;F1KQP6;U1MGQ2	F1KQK4	826	847	22	-	-	-	0.0001	Female	Medium			VLPMDAETM	VLPMDAETM			
SYRNIIQTLYGGQLEQ	F1L2I5	F1L2I5	134	149	16	-	-	-	0.00012	Female	Medium			IQTLYGGQL	IQTLYGGQL			
QILPYKEIPGFPNT	F1LBA4;U1MEL8	F1LBA4	58	71	14	-	-	-	0.00014					YKEIPGFPN	ILPYKEIPG			
ADFTAYRAFTPIPK	F1L859	F1L859	384	397	14	-	-	-	3.34E-05			<=WB	<=WB	YRAFTPIPK	YRAFTPIPK			
AKVKEYFNELPAEKQ	F1KTI9;F1KUF9	F1KTI9	237	251	15	-	-	-	1.45E-05	Female	High			FNELPAEKQ	VKEYFNELP			
VEEKVEQQEQTE	U1M0J2	U1M0J2	30	41	12	-	-	-	1.67E-05					EKVEQQEQT	EKVEQQEQT			
DELRKADNELTDDLEKYHRS	F1KQS3;U1M491	F1KQS3	1572	1591	20	-	6.79E-06	-	0.00205	Female	Medium			LRKADNELT	LRKADNELT			
**LRQIAQAIVAPGKGILAADEST**	**F1L5H2;U1M5S0**	**F1L5H2**	**27**	**48**	**22**	**-**	**8.54E-06**	**-**	**0.00059**	**No**	**High**			**AIVAPGKGI**	**VAPGKGILA**			**IAQAIVAPGKGILAA**
AVDMMWINNYFPYYANDK	F1L2I5	F1L2I5	224	241	18	-	3.83E-06	-	0.00052	Female	Medium			MWINNYFPY	INNYFPYYA			
IIVPNEGDVTAFKNYEASGGGIA	U1LYB7	U1LYB7	628	650	23	-	-	-	0.00115					YEASGGGIA	VTAFKNYEA			
TLIDYMSSGPVVA	F1LG83	F1LG83	111	123	13	-	-	-	0.00086	Female	Medium	<=WB	<=WB	IDYMSSGPV	IDYMSSGPV			
GMNVVREIESTPTHPGDRPQ	F1LEZ4;U1NRW8	F1LEZ4	203	222	20	-	-	-	0.00086	Female	Medium			IESTPTHPG	VREIESTPT			
IENNQLVPDPIDHLETWKVLEK	F1KVX7;F1LB68	F1KVX7	40	61	22	-	-	-	0.00089	No	Medium			IDHLETWKV	IDHLETWKV			
EIEADIDAFVAKLGADYTNK	F1LAR2;Q86M44	F1LAR2	99	118	20	-	-	-	0.00091	No	Medium			FVAKLGADY	FVAKLGADY			
ALHAVTDEEKKQYIADFGPA	F1KTI9;F1KUF9;Q06811	F1KTI9	863	882	20	-	-	-	0.00102	Female	High			QYIADFGPA	KKQYIADFG			
FGEILDYLEKNLKIK	F1KPM2;U1NWH3	F1KPM2	2383	2397	15	-	-	-	0.00097	Female	Medium			DYLEKNLKI	ILDYLEKNL		+	
LTEPVDEYTIQAMPEFDGKRFQN	F1KUF1;F1KV29	F1KUF1	590	612	23	-	-	-	0.00096	No	Low			YTIQAMPEF	IQAMPEFDG			
ATDFLDQQFALIGTFANKRGGTAL	F1KPM2;U1NWH3	F1KPM2	2633	2656	24	-	-	-	0.00022	Female	Medium		<=WB	FALIGTFAN	FALIGTFAN		+	
ALEIAKEQGINVFEG	F1LBA4;U1MEL8	F1LBA4	187	201	15	-	-	-	0.00021					IAKEQGINV	IAKEQGINV			
AQKQLVKYLLSTESDAATYTE	F1KPM2;U1NWH3	F1KPM2	2101	2121	21	-	-	-	0.0006	Female	Medium			VKYLLSTES	VKYLLSTES			
SSLLQPNDVCSVVAQESHGTVF	F1KPM2;U1NWH3	F1KPM2	3048	3069	22	-	-	-	0.0006	Female	Medium			VVAQESHGT	VVAQESHGT			
YTDDIIVYTPK	F1KXK9	F1KXK9	189	199	11	-	-	-	0.00051	Female	Medium			DDIIVYTPK	DDIIVYTPK			
NSNAQEVVLGPAPHLVYRTIGGNID	F1KUA0	F1KUA0	50	74	25	-	-	-	0.00033	Male	High			VVLGPAPHL	VVLGPAPHL			
DRNGNYLWPGYGENIRVIDW	F1L041;U1MDY4	F1L041	596	615	20	-	-	-	0.00033	Female	Medium			LWPGYGENI	LWPGYGENI			
DSADKTLANFGWGGQENAG	Q7YXK2	Q7YXK2	235	253	19	-	-	-	0.00026	Female	Medium			TLANFGWGG	TLANFGWGG			
RNNVLKNLLTSFLQQVKN	U1LTC3	U1LTC3	382	399	18	-	-	-	0.00027	Female	Medium			LKNLLTSFL	LKNLLTSFL		+	
DDLEQYSRSY	F1KQK4;U1MGQ2	F1KQK4	1624	1633	10	-	-	-	0.00028	Female	Medium			DLEQYSRSY	DLEQYSRSY			
GNDISKGEVLSEYISSAPPPNTG	F1L5C6;F1LC55	F1L5C6	116	138	23	-	-	-	0.00029	No	Medium			YISSAPPPN	YISSAPPPN			
RTVVITFEKMNNME	F1L5N8;F1LHJ8;U1NI43	F1L5N8	233	246	14	-	-	-	0.00044					VVITFEKMN	VITFEKMNN			
GTVYDMGSRDLNNL	U1NYZ1	U1NYZ1	113	126	14	-	-	-	0.00039	Female	Medium			VYDMGSRDL	VYDMGSRDL			
VFGTLNTLVKSWLNEQRE	F1KPM2;U1NWH3	F1KPM2	2273	2290	18	-	-	-	0.00038	Female	Medium			FGTLNTLVK	LNTLVKSWL			
YGEENEVGNIEPYLL	F1LHW8;U1MA99	F1LHW8	54	68	15	-	-	-	0.00038	No	Medium			VGNIEPYLL	VGNIEPYLL			
YVALWYKHGQPVFGRAYPD	Q7YXK2	Q7YXK2	217	235	19	-	-	-	0.00041	Female	Medium			YKHGQPVFG	LWYKHGQPV			
ASNIVSYGERPNVKK	A8Y5V9;F1L7R9	A8Y5V9	127	141	15	4.98E-05	-	0.02744	6.71E-06	Male	Medium			IVSYGERPN	IVSYGERPN			
STFPDEQPRIVVIPYAFFLGAVA	U1MRA1	U1MRA1	554	576	23	0.00037664	-	0.00274	0.00012	No	High			YAFFLGAVA	YAFFLGAVA			
THGSAQSPININIANVVAQKFDE	F1L837	F1L837	64	85	23	0.00264284	-	0.00099	0.00015	No	Medium			ININIANVV	ININIANVV	+		
VITTYSSFLSPVS	F1KPL9	F1KPL9	1817	1829	13	0.00026564	1.84E-05	0.00168	1.77E-05	Male	Medium	<=WB	<=WB	YSSFLSPVS	ITTYSSFLS	+		
AKSMGVSVGIYTNYYNWQEIVG	F1LE63;F1LEA7	F1LE63	142	158	22	7.62E-05	-	0.00078	1.13E-05	Male	High			MGVSVGIYT	VGIYTNYYN			
ILLYTSAYGEGGFTDPKAIANQIKE	F1L0R7	F1L0R7	295	319	25	0.00027067	-	0.00071	2.11E-05	No	Medium			LLYTSAYGE	ILLYTSAYG			
YRVFGTIQNFPAFR	F1KPL9;F1KQT8;U1MQT5	F1KPL9	676	689	14	0.00014107	-	0.00043	1.70E-05	Male	Medium	<=WB	<=SB	FGTIQNFPA	FGTIQNFPA	+		
TLNMMQYANARIF	F1KPL9	F1KPL9	1114	1126	13	0.00024568	-	0.00026	8.33E-06	Male	Medium	<=WB	<=WB	MQYANARIF	MMQYANARI	+		
SREAPKLVASLSNLNLVEGQT	F1KQG0	F1KQG0	923	943	21	1.12E-05	-	7.78E-05	-			<=WB	<=WB	LVASLSNLN	LVASLSNLN			
SLYGHSVDALGMLVSTDASGK	F1KPH3;F1KPH7	F1KPH3	2821	2841	21	9.45E-06	-	3.13E-05	-	No	Low			GHSVDALGM	LGMLVSTDA			
LGGPIFFYTGNEGSIEE	F1L108;U1MJ81	F1L108	81	97	17	6.92E-05	-	5.24E-05	-	Male	Medium			FFYTGNEGS	IFFYTGNEG			
TKDTSQGSNGNALNPYGEDISPLKF	U1P3P5	U1P3P5	102	126	25	4.17E-05	-	0.00237	-	Male	High			YGEDISPLK	LNPYGEDIS			
SELAFGRAKTVDGGFAPSGKNIYK	F1LDH6	F1LDH6	40	63	24	5.86E-05	-	0.00043	-	Male	Medium			FAPSGKNIY	FAPSGKNIY			
FWIGAYENNGVYTWPDGSK	A8Y5V9;F1L7R9;U1N9J0	A8Y5V9	295	313	19	2.70E-05	-	0.00035	-	Male	Medium			IGAYENNGV	IGAYENNGV			
STSRPTAIWYKDGVPIRE	F1KPE9;F1KPK6;U1MCT2	F1KPE9	136	153	18	6.65E-05	-	0.00055	-	Male	Low			IWYKDGVPI	IWYKDGVPI			

Supplementary Table 2 (cont).

EDGRTLSDYNIQ	F1L201;F1L737;F1L9Q8;F1LAS0;F1LBR8;F1LDA0;F1LDJ7;F1LEI3;F1LEJ6;F1LEJ7;F1LGK4;F1LGV3;F1LHW4;U1M1W1;U1M349	F1L201	68	79	12	-	-	0.00139	-	No	Medium			GRTLSDYNI	GRTLSDYNI			
FIDWEEYLRQLSTYADTSVRKQ	F1KPL9	F1KPL9	1755	1776	22	-	-	0.00101	-	Male	Medium			YLRQLSTYA	YLRQLSTYA		+	
AARNQNVLDWNGNLLNVQE	F1KR19	F1KR19	1104	1122	19	-	-	0.00035	1.28E-05					LDWNGNLLN	VLDWNGNLL			
LSMLGRDIIAYAGQPAK	F1KPE9;U1MCT2	F1KPE9	4920	4935	17	-	-	0.00059	5.36E-06	Male	Low		<=WB	IIAYAGQPA	IIAYAGQPA			
TTYPALVNYFNAANRAF	F1KPL9	F1KPL9	1013	1028	17	-	-	0.00015	2.67E-06	Male	Medium		<=WB	YFNAANRAF	LVNYFNAAN			
GNLPADIVTYLGERGNGQY	F1KR19	F1KR19	1441	1459	19	-	-	6.81E-05	4.61E-06					IVTYLGERG	IVTYLGERG			
DKYIQLATYFPSSNVYG	U1P3P5	U1P3P5	202	218	17	-	-	0.00033	-	Male	High			IQLATYFPS	IQLATYFPS			
AALGKYTGGGAGSAAGQS	F1L5H2;F1LCU9;U1M5S0	F1L5H2	348	365	18	-	-	9.74E-05	-	No	High			YTGGGAGSA	LGKYTGGGA			
VTELGRPDAEY	DRB*01:15	DRB*01:15	104	114	11	-	-	0.0001	-					VTELGRPDA	VTELGRPDA			
DDEETFHMY	F1L771;P28316	F1L771	247	255	9	-	-	0.00013	-	No	Medium			DDEETFHMY	DDEETFHMY			
VQVTTYYNYPYVPDLPTQHEDKE	F1KQN0	F1KQN0	418	440	23	-	-	0.00013	-	No	Medium			VTTYYNYPY	VTTYYNYPY			
AFVNGNLDDFTSYNSL	F1L0R7	F1L0R7	241	256	16	-	-	0.00013	-	No	Medium			LDDFTSYNS	LDDFTSYNS			
FEEAVLASYDTAQQAVEQA	F1KPM2;U1NWH3	F1KPM2	2313	2331	19	-	-	7.48E-05	-	Female	Medium			LASYDTAQQ	LASYDTAQQ			
TIVNYLNFRVIDT	F1KPL9	F1KPL9	297	309	13	-	-	1.72E-05	-	Male	Medium		<=SB	VNYLNFRVI	IVNYLNFRV		+	
VIDIFGYDWLPH	F1KX94	F1KX94	301	312	12	-	-	2.12E-05	-	Male	High			IFGYDWLPH	IFGYDWLPH			
ATYDKGTVTILNDQHIR	U1MIR8	U1MIR8	146	162	17	-	-	4.02E-05	-	No	Medium			YDKGTVTIL	VTILNDQHI		+	
DTTIVSIQEGGTVK	F1KPF4;F1KPG3	F1KPF4	1749	1762	14	-	-	4.40E-05	-	Male	Medium			VSIQEGGTV	VSIQEGGTV			
ANAGDYIITIRNPTGAAQ	F1KPJ7;F1KPK1	F1KPJ7	3186	3203	18	-	-	4.04E-05	-	No	Low			IITIRNPTG	IITIRNPTG			
PPAANMPAGGGVSTHTLTNPSAVRL	Q7YXK3	Q7YXK3	62	86	25	-	0.0009091	3.25E-06	-					HTLTNPSAV	TLTNPSAVR			
NAALKNTIVPMVE	F1KPM2;U1NWH3	F1KPM2	2252	2264	13	2.06E-06	0.0037937	-	-	Female	Medium			LKNTIVPMV	LKNTIVPMV	+		
LIEWTPANVNWVYE	F1LG83	F1LG83	187	200	14	-	0.0014014	-	-	Female	Medium	<=WB		IEWTPANVN	IEWTPANVN			
TNGIEKTNDAVIVE	F1KQK4	F1KQK4	1562	1575	14	-	0.0012058	-	-	Female	Medium			IEKTNDAVI	IEKTNDAVI			
YGDAATHPIDFGIAPASVIPKV	F1KYX0	F1KYX0	301	322	22	-	0.0012212	-	-	No	Medium	<=WB		FGIAPASVI	FGIAPASVI			
TGVLLSRSVILTAGHGFLNEKGEAIR	U1NQU8	U1NQU8	99	124	26	-	0.0039049	-	-	Female	Medium	<=WB		VLLSRSVIL	VLLSRSVIL			
**EAEYEKAHAAAIAKFSPA**	**F1LAR2;Q86M44**	**F1LAR2**	**129**	**143**	**18**	**-**	**0.0029771**	**-**	**-**	**No**	**Medium**	**<=WB**		**YEKAHAAAI**	**YEKAHAAAI**	**+**		**KAHEAEYEKAHAAAIAKFSP**
IRELIEDVAALPMPV	F1LA15;U1P0Q2	F1LA15	138	152	15	-	0.0033971	-	-	No	Medium			IEDVAALPM	IEDVAALPM			
AVEVRNYIKSKVDSLGPEAK	F1LEI7	F1LEI7	87	106	20	-	0.0002329	-	2.38E-06	Female	Medium			YIKSKVDSL	VRNYIKSKV			
LNEAISITNRLDE	U1LTC3	U1LTC3	344	356	13	-	0.000367	-	4.62E-06	Female	Medium			EAISITNRL	AISITNRLD			
DIAKDKMQPTHPIRL	F1KXW6;F1LAU0;F1LF96;W6EA75	F1KXW6	159	173	15	-	0.0003658	-	-	Female	High			MQPTHPIRL	MQPTHPIRL			
NAGKDTNGSQFFITTVQTSWLNGRH	F1LEZ4;U1NRW8	F1LEZ4	170	194	25	-	0.0003478	-	-	Female	Medium			FFITTVQTS	ITTVQTSWL			
TERGYSFTTTAEREIVRDIKE	F1L3U5;F1LAW8;F1LD26;F1LH12;U1NG85	F1L3U5	255	275	21	-	0.000245	-	-	No	Medium			YSFTTTAER	YSFTTTAER			
APLGEEDVAMRSVVVGRLE	F1KQS3;U1M491	F1KQS3	296	314	19	-	0.0002746	8.38E-07	-	Female	Medium			DVAMRSVVV	DVAMRSVVV			
RGGTALESIKAEILGDEIAIH	U1NWH3	U1NWH3	2633	2653	21	-	0.0002799	-	-	Female	Medium			LESIKAEIL	LESIKAEIL			
GALLGELTLPL	Q6BD87;Q6BD89;Q6BD92;Q6BD94;Q6BD96;Q8ISC1	Q6BD87	146	156	11	-	0.0002794	-	-					LLGELTLPL	LLGELTLPL			
FDGQVASVKVPLL	U1MGQ2	U1MGQ2	1529	1541	13	-	0.0002796	-	-					VASVKVPLL	VASVKVPLL			
VRATSSNELGVEPEA	F1KQK4;F1KQP6;F1L7H2;U1MGQ2	F1KQK4	384	398	15	-	0.0006564	-	-	Female	Medium			VRATSSNEL	VRATSSNEL			
PPAMDVLKGDAADMSPSQPKQTD	F1KSR7	F1KSR7	678	700	23	-	0.0007454	-	-					VLKGDAADM	VLKGDAADM			
VQEWVNSQDSSVQ	U1M8L9	U1M8L9	71	83	13	-	0.0005809	-	-	Female	Low			WVNSQDSSV	WVNSQDSSV			
AGQDATSVLKTLAPHV	Q17091	Q17091	78	93	16	-	0.000515	-	-					VLKTLAPHV	VLKTLAPHV			
DQMGENSRDTYGQIMGSRL	U1LTC3	U1LTC3	451	469	19	-	0.0005186	-	-	Female	Medium			YGQIMGSRL	YGQIMGSRL			
NGSNENEATYADMFYEGIRDLHM	F1L8V4;U1MKZ2	F1L8V4	37	59	23	-	0.0001745	-	-	No	Medium			YEGIRDLHM	MFYEGIRDL			
STNLKDVLADKIPQ	F1KSX9;F1KWZ6;U1MH21	F1KSX9	31	44	14	-	0.0001312	-	-					LKDVLADKI	LKDVLADKI			
NLGISNVNAAKL	F1KZY8;F1LCS2	F1KZY8	75	86	12	-	0.0001354	-	-	Female	Medium	<=WB		ISNVNAAKL	ISNVNAAKL			
NISNSIPVIRG	F1KSI5;U1N8L0	F1KSI5	24	34	11	-	7.67E-05	-	-	Female	Medium			ISNSIPVIR	ISNSIPVIR			
GGASDREFNEHFSKLL	F1L2I5	F1L2I5	90	105	16	-	9.68E-05	-	-	Female	Medium			FNEHFSKLL	FNEHFSKLL			
TIHPLDNLSMHPAAKVL	F1LCS2	F1LCS2	84	100	17	-	8.67E-05	-	-	Female	Medium	<=WB		LSMHPAAKV	LSMHPAAKV			
YSQSVKIL	F1LBE9	F1LBE9	118	125	8	3.06E-06	2.11E-05	-	-	Female	Medium							
YTDDPSRPEDEWQITRVGSPDV	F1KPQ8	F1KPQ8	581	602	22	8.59E-06	8.33E-06	-	-	No	Medium			ITRVGSPDV	ITRVGSPDV	+		
AFKGTSDMPLIQNF	U1NPX4	U1NPX4	1643	1656	14	1.16E-05	4.15E-05	-	-	No	Medium	<=WB		FKGTSDMPL	FKGTSDMPL	+		
HKDDYGAFKLSHGKFYNDAVKDK	F1L144;F1L1U2;F1L878;F1LBI5	F1L144	39	61	23	-	3.74E-05	-	-	Female	Medium	<=WB		FKLSHGKFY	FKLSHGKFY			
VKQLQSSASQLLSVQNGLNL	F1L7Z3	F1L7Z3	330	349	20	-	3.85E-05	-	-			<=WB		LQSSASQLL	LQSSASQLL			
YQSDPSGNYTGWKATCIGNNHQ	F1LD64;F1LDS6	F1LD64	148	169	22	-	4.58E-05	-	-					YTGWKATCI	YTGWKATCI			
LSQPNKSYKMSSGNKV	F1L2I5	F1L2I5	108	123	16	-	5.95E-05	-	-	Female	Medium	<=SB		YKMSSGNKV	YKMSSGNKV			
LPYLAHSPNEIEAMLSG	F1LB68	F1LB68	82	98	17	-	5.78E-05	-	-	No	Medium			YLAHSPNEI	YLAHSPNEI			
VAPVEITRTAGPP	F1LI20;Q7YXK3	F1LI20	56	68	13	-	2.87E-05	-	-	No	Medium			VEITRTAGP	VEITRTAGP			
SQLIWSRAMGLPIERPK	U1MH21	U1MH21	461	477	17	-	3.10E-05	-	-			<=SB	<=WB	WSRAMGLPI	LIWSRAMGL			
RLIGNTNAFLIKDVDLK	F1KQK4;F1KQP6;U1MGQ2	F1KQK4	277	293	17	-	2.59E-05	-	-	Female	Medium			IGNTNAFLI	LIGNTNAFL			
SSGLSDLYDPQNSYMTFEGVQVKGR	F1LDS9	F1LDS9	31	55	25	-	2.39E-05	-	-					YMTFEGVQV	YMTFEGVQV			
EWESPKGVPID	F1KSI5;F1KXJ0;F1KYG6;U1N8L0	F1KSI5	437	447	11	-	1.92E-05	-	-	Female	Medium			WESPKGVPI	WESPKGVPI			
SPVGRYLTTGFAPITL	F1KZY8;F1LCS2	F1KZY8	91	106	16	-	1.50E-05	-	-	Female	Medium	<=WB		YLTTGFAPI	YLTTGFAPI			
FVTSTSSWPQNTK	F1KQK4;F1KQP6;U1MGQ2	F1KQK4	1000	1012	13	-	1.49E-05	-	-	Female	Medium			VTSTSSWPQ	VTSTSSWPQ			
**VELTGARDLGGQIQ**	**U1MSR9**	**U1MSR9**	**64**	**77**	**14**	**-**	**1.47E-05**	**-**	**-**					**VELTGARDL**	**LTGARDLGG**			**NKVELTGARDLGGQI**
GDYSIQTGRNIVHGSDS	F1LG83	F1LG83	154	170	17	-	1.13E-05	-	-	Female	Medium			YSIQTGRNI	YSIQTGRNI			
DSYTKSGSKPSEL	F1LHI5	F1LHI5	108	120	13	-	2.90E-06	-	-					YTKSGSKPS	YTKSGSKPS			
TFTTQETITNAESAK	F1KUW6;U1NWT5	F1KUW6	233	247	15	-	1.74E-06	-	-	No	Medium			FTTQETITN	FTTQETITN			
SQVVQSGAQNIE	F1L4Z3;F1LAK0;F1LCI3;F1LDC1;U1NAA7	F1L4Z3	127	138	12	-	2.43E-06	-	-					VVQSGAQNI	VVQSGAQNI			
VEEALHAVTDEEKK	F1KTI9;F1KUF9;Q06811	F1KTI9	860	873	14	-	3.68E-06	-	-	Female	High			LHAVTDEEK	LHAVTDEEK			
SWELTGDEKLE	F1L8L6;U1N6X9	F1L8L6	243	253	11	-	3.82E-06	-	-	Female	Medium			WELTGDEKL	WELTGDEKL			
LYKSGVYQHVTGEMMGGHAI	P07858	P07858	261	280	20	-	4.13E-06	-	-					YQHVTGEMM	YQHVTGEMM			
**ITDSIEEAKSFAAE**	**F1L144;F1L1U2;F1L878;F1LBI5**	**F1L144**	**329**	**342**	**14**	**-**	**4.26E-06**	**-**	**-**	**Female**	**Medium**			**IEEAKSFAA**	**IEEAKSFAA**			**TDSIEEAKSFAAETF**
AVVKATEAGAVT	F1L2P3;F1L9F1	F1L2P3	356	367	12	-	4.41E-06	-	-	No	Medium			VKATEAGAV	VKATEAGAV			
ATTGDIVNTSSVSAVTASK	F1LAI9;U1LRQ7	F1LAI9	138	156	19	-	5.58E-06	-	-					IVNTSSVSA	IVNTSSVSA			
GAVKPWQEAGGHHISE	F1KWJ7;F1L1T0;F1L5I9;F1LFT3;U1MDM8	F1KWJ7	251	266	16	-	5.07E-06	-	-					WQEAGGHHI	WQEAGGHHI			
LAEFNDAFSKLISESSLSDEAKL	U1M683	U1M683	74	96	23	-	5.28E-06	-	-					FNDAFSKLI	FSKLISESS			
FYETYHKMATTIGIPLAFS	F1KQK4;F1KQP6;U1MGQ2	F1KQK4	879	897	19	-	6.56E-06	-	-	Female	Medium	<=WB		MATTIGIPL	MATTIGIPL			
**RSENKQSLIYSPESLIK**	**F1KQS3;U1M491**	**F1KQS3**	**329**	**345**	**17**	**-**	**6.32E-06**	**-**	**-**	**Female**	**Medium**			**LIYSPESLI**	**LIYSPESLI**			**KQSLIYSPESLIKIE**
VGIDMSHSSSQSL	F1KS82	F1KS82	702	714	13	-	7.53E-06	-	-					MSHSSSQSL	IDMSHSSSQ			
ATQLNEYKIVASYEVR	F1KQS3;U1M491	F1KQS3	1316	1331	16	-	7.89E-06	-	-	Female	Medium	<=SB	<=WB	YKIVASYEV	YKIVASYEV			
IVKGHSSKYETFRPHVV	F1LHI5	F1LHI5	72	88	17	-	8.34E-06	-	-					YETFRPHVV	YETFRPHVV			
ELTRMILVNAVYFSGTWKNIFKK	F1L2I5	F1L2I5	185	207	23	-	8.52E-06	-	-	Female	Medium			VYFSGTWKN	VYFSGTWKN			
LEKSYELPDGQVIT	F1L3U5;F1LAV8;F1LAW4;F1LAW5;F1LAW8;F1LAW9;F1LBK2;F1LCH4;F1LCH5;F1LD26;F1LDN8;F1LDP8;F1LEQ3;F1LET7;F1LGL4;F1LGL5;F1LH12;F1LHF1;U1M7Z4;U1MLY3;U1MSU7;U1NG85	F1L3U5	297	310	14	-	8.77E-06	-	-	No	Medium			YELPDGQVI	YELPDGQVI			
GSHSITQSELLDS	F1KPM2;U1NWH3	F1KPM2	2992	3004	13	-	8.78E-06	-	-	Female	Medium			HSITQSELL	HSITQSELL			
ITPVTVFDKGQYTIVAVNTLGESR	F1KQG0	F1KQG0	993	1011	24	0.0030384	2.68E-06	2.86E-05	-					YTIVAVNTL	YTIVAVNTL	+		
AQILPIDDNKLKVEWLHNGSPLK	F1KPJ7;F1KPK1	F1KPJ7	147	169	23	0.00401791	-	3.07E-05	-	No	Low			WLHNGSPLK	ILPIDDNKL			
AASFVKTIPVSQAIG	A8Y5V9;F1L7R9	A8Y5V9	60	70	15	0.00176521	-	0.00012	-	Male	Medium	<=WB		FVKTIPVSQ	VKTIPVSQA	+	+	
YVFRYSGAGPLNGDPDVILRGN	F1KPF4;F1KPG3;F1KPK5	F1KPF4	940	961	22	5.69E-05	-	-	9.57E-06	Male	Medium	<=SB		FRYSGAGPL	FRYSGAGPL	+	+	
SYLNIIRSIYDGQLEQ	F1L1S5	F1L1S5	134	149	16	2.03E-05	-	-	6.59E-05	No	High			LNIIRSIYD	LNIIRSIYD	+	+	
YVYDVTSTASLQLMQGK	F1KPM2;U1NWH3	F1KPM2	57	73	17	0.00010117	3.30E-06	-	-	Female	Medium	<=WB		VTSTASLQL	VTSTASLQL	+		
DTGDTQLGPVSAEEIK	F1LGC3	F1LGC3	18	33	16	0.00014541	3.53E-06	-	-					LGPVSAEEI	LGPVSAEEI	+		
VGFIHTSLSEVNMHFSDIYKQ	U1P3X1	U1P3X1	209	229	21	0.00012523	1.81E-05	-	-	Male	Medium			FIHTSLSEV	VNMHFSDIY	+		
EEDMTKFKPDFTDITAENIITF	B2REF9;F1LCN6;F1LEG5	B2REF9	326	338	22	5.24E-05	7.89E-06	-	-	Female	Medium			FTDITAENI	FTDITAENI	+		
YGKITDTVVNVISPPF	F1KQT8;U1MQT5	F1KQT8	1243	1258	16	5.85E-05	1.51E-05	-	-	Male	Medium			VVNVISPPF	VVNVISPPF	+		
FIPFGSNEIQE	F1LF53;Q6PWM9	F1LF53	87	97	11	3.67E-05	-	-	-	Male	Medium			FIPFGSNEI	FIPFGSNEI	+		
KGPLLVTTEETVNE	F1KPF4;F1KPG3;F1KRA2	F1KPF4	2591	2604	14	3.69E-05	-	-	-	Male	Medium			LLVTTEETV	LLVTTEETV			
LAYQTHGWSKSTDWAIASLTNLVTE	F1KQF7;U1NL82	F1KQF7	1533	1557	25	3.14E-05	-	-	-	No	Medium			WSKSTDWAI	IASLTNLVT			
SVIDNFKAASGLEFS	F1KQT8;U1MQT5	F1KQT8	908	922	15	2.80E-05	-	-	-	Male	Medium	<=SB		FKAASGLEF	FKAASGLEF	+		
NEWRGSDSETIGAIADE	F1KPH3;F1KPH7	F1KPH3	3582	3598	17	2.35E-05	-	-	-	No	Low			WRGSDSETI	WRGSDSETI			
YSGLNKHVPAVYIENDE	U1M5W7	U1M5W7	195	211	17	2.32E-05	-	-	-	Male	Medium			NKHVPAVYI	LNKHVPAVY			
FAQTIDHYVNSGGGPYI	F1KSB9;U1NBY1	F1KSB9	741	757	17	2.45E-05	-	-	-					YVNSGGGPY	IDHYVNSGG			
NDEELHESTDVHI	F1KPF2;U1NFY6	F1KPF2	608	620	13	1.89E-05	-	-	-					LHESTDVHI	LHESTDVHI			
AQLEWRREDGNALNDEATDE	F1KPF4;F1KPG3;F1KRA2	F1KPF4	2248	2267	20	1.93E-05	-	-	-	Male	Medium			WRREDGNAL	WRREDGNAL			
KPLADTTIVSIQE	F1KPF4;F1KPG3	F1KPF4	1745	1757	13	2.13E-05	-	-	-	Male	Medium			LADTTIVSI	LADTTIVSI			
YVGYITMGSQRTPM	U1NHZ7	U1NHZ7	80	93	14	2.10E-05	-	-	-			<=WB	<=WB	YITMGSQRT	YITMGSQRT			
SEEFEFSILEPAMLTK	F1KQT8	F1KQT8	332	347	16	2.18E-05	-	-	-	Male	Medium	<=WB		FSILEPAML	FSILEPAML	+		
VWQKPWENFEQFTIKE	F1KVP6	F1KVP6	118	133	16	1.30E-05	-	-	-					WENFEQFTI	WENFEQFTI			
YELSSANKL	F1L040;F1L0N2;F1L6X4;U1M2W9	F1L040	43	51	9	1.37E-05	-	-	-	No	Medium	<=SB	<=WB	YELSSANKL	YELSSANKL	+		
FVESQGNPASDPVV	F1KPY0	F1KPY0	1425	1438	14	1.01E-05	-	-	-	Male	Medium			FVESQGNPA	FVESQGNPA			
SYRPVSNPYGDNISPIF	F1KPW3;F1KRB6;F1KX94	F1KPW3	1363	1379	17	9.97E-06	-	-	-	Male	High			YRPVSNPYG	YRPVSNPYG			
EQIANAQVTIHPTNGGPL	F1KPF4;F1KPG3;F1KUT4	F1KPF4	4070	4087	18	1.14E-05	-	-	-	Male	Medium			IHPTNGGPL	IHPTNGGPL			
FDGKSYDMAVALWIPDSDK	U1M5W7	U1M5W7	110	128	19	1.16E-05	-	-	-	Male	Medium			YDMAVALWI	YDMAVALWI			
YTAAIFAVVAHIVANTED	F1L4G6;U1NNG2	F1L4G6	438	455	18	6.03E-06	-	-	-					IFAVVAHIV	IFAVVAHIV			
SLLAQSSGYTVR	U1MRA1	U1MRA1	82	93	12	6.81E-06	-	-	-	No	High	<=WB		LAQSSGYTV	LLAQSSGYT			
LIVNSNAQEL	F1KPW3	F1KPW3	619	628	10	8.35E-06	-	-	-	Male	High			IVNSNAQEL	IVNSNAQEL	+		
TYKVIMNNYLVDRHQ	F1KQT8;U1MQT5	F1KQT8	1295	1309	15	8.95E-06	-	-	-	Male	Medium	<=WB	<=SB	YKVIMNNYL	YKVIMNNYL	+		
QGAGENIYQFSTTGRIPSTE	F1LDH6	F1LDH6	92	111	20	7.77E-06	-	-	-	Male	Medium	<=WB		YQFSTTGRI	IYQFSTTGR	+		
DYYIRLQPYFPVPG	U1NL82	U1NL82	73	86	14	5.13E-06	-	2.66E-06	-	No	Medium	<=WB	<=SB	IRLQPYFPV	IRLQPYFPV	+	+	
WTKVGTNTLPDSA	F1KPF4;F1KPG3;F1KPK5	F1KPF4	1296	1308	13	3.68E-06	-	-	-	Male	Medium			WTKVGTNTL	WTKVGTNTL			
SNLTENIQKIQEDIDE	U1P3X1	U1P3X1	285	300	16	4.33E-05	-	-	-	Male	Medium			NLTENIQKI	LTENIQKIQ			
ATVQFSNSRLL	F1KQV9	F1KQV9	1248	1258	11	5.35E-05	-	-	-			<=SB	<=WB	VQFSNSRLL	VQFSNSRLL			
MNQTYTDAQKLE	U1MJD0	U1MJD0	206	217	12	5.54E-05	-	-	-	Male	Medium			QTYTDAQKL	QTYTDAQKL			
AAKAAFLKRAQANGLAALGK	F1L5H2;F1LCU9;U1M5S0	F1L5H2	333	352	20	5.19E-05	-	-	-	No	High	<=WB		LKRAQANGL	LKRAQANGL	+		
ALNDFQTTTGLTF	F1KPL9	F1KPL9	842	854	13	5.08E-05	-	-	-	Male	Medium	<=WB		FQTTTGLTF	FQTTTGLTF			
ANRDTHFTTADKIGILGDE	F1KQF7;U1NL82	F1KQF7	548	566	19	4.76E-05	-	-	-	No	Medium			FTTADKIGI	FTTADKIGI			
AVNEFGKASSEAML	F1KPF2;U1NFY6	F1KPF2	1672	1685	14	4.85E-05	-	-	-					FGKASSEAM	FGKASSEAM			
ISGIQDFSMFGIPYVG	F1KX94	F1KX94	42	57	16	4.93E-05	-	-	-	Male	High			FSMFGIPYV	FSMFGIPYV			
LAEYGISNADNILTRE	F1LDH6	F1LDH6	125	140	16	0.00015105	-	-	-	Male	Medium			YGISNADNI	ISNADNILT			
LDISSTDSYTT	F1KPL9	F1KPL9	1965	1975	11	0.00013036	-	-	-	Male	Medium			ISSTDSYTT	ISSTDSYTT			
LIDPLYATTSDYSIVAR	U1NPX4	U1NPX4	1310	1326	17	0.00013814	-	-	-	No	Medium			YATTSDYSI	LYATTSDYS			
RQDGSSFGYDITDEDGILTIPR	F1KPF4;F1KPG3	F1KPF4	2068	2089	22	0.0001094	-	-	-	Male	Medium			YDITDEDGI	ITDEDGILT			
SREGEEDLPDNAQVTGNRL	F1KPF4;F1KUT4	F1KPF4	4305	4323	19	0.00011137	-	-	-	Male	Medium			NAQVTGNRL	NAQVTGNRL			
LQQFQLATTLSFPLLEQS	F1KPL9	F1KPL9	1601	1618	18	9.94E-05	-	-	-	Male	Medium	<=SB		FQLATTLSF	LATTLSFPL	+		
SVGEMEFAQTIDHYVNSGGGPY	F1KSB9;U1NBY1	F1KSB9	735	756	22	0.00010304	-	-	-					FAQTIDHYV	FAQTIDHYV			
VYSLYKQATVGDINTPKPGATD	F1LD21;U1P442	F1LD21	81	102	22	9.62E-05	-	-	-					YKQATVGDI	YSLYKQATV			
LSNDQGTYLDFASLRDL	F1KPP1;U1MB48	F1KPP1	2766	2782	17	7.36E-05	-	-	-					YLDFASLRD	YLDFASLRD			
SVYAMQFATGRIF	F1KQT8;U1MQT5	F1KQT8	1183	1195	13	8.90E-05	-	-	-	Male	Medium	<=WB	<=WB	MQFATGRIF	AMQFATGRI	+		
GQIEPTNDNRLT	F1KPJ7;F1KPK1	F1KPJ7	545	556	12	8.44E-05	-	-	-	No	Low			IEPTNDNRL	IEPTNDNRL			
IDSSLSMTKEGLIQ	F1L8I9;U1NRL6	F1L8I9	53	66	14	8.33E-05	-	-	-					LSMTKEGLI	LSMTKEGLI			
DPWIYQATYPVLK	F1KQL5;F1KR19;F1KW74;U1M1R7	F1KQL5	1413	1423	13	0.00055927	-	9.86E-06	-	No	High	<=SB	<=WB	WIYQATYPV	WIYQATYPV	+	+	
LMDPWIYQATYPV	F1KQL5;F1KR19;F1KW74;U1M1R7	F1KQL5	1413	1423	13	0.00055927	-	9.86E-06	-	No	High	<=WB		WIYQATYPV	WIYQATYPV			
**YRYLPYLYTLHYFAATSGSTVVR**	**F1KX94**	**F1KX94**	**116**	**138**	**23**	**0.00030969**	**4.28E-06**	**3.71E-05**	**-**	**Male**	**High**	**<=SB**	**<=WB**	**FAATSGSTV**	**LHYFAATSG**	**+**	**+**	**LHYFAATSGSTVVRP**

Supplementary Table 2 (cont).

LSNDFSTFDSNMVANYF	F1KPL9	F1KPL9	1040	1055	17	0.00024564	-	3.59E-05	-	Male	Medium	<=WB		FSTFDSNMV	FSTFDSNMV	+	+	
SSFSTASTRNIIK	F1KPL9	F1KPL9	26	38	13	0.00033722	2.19E-06	1.23E-05	-	Male	Medium	<=SB		FSTASTRNI	FSTASTRNI	+		
FSMVYSQASQSPQSLDK	F1KPL9	F1KPL9	2368	2384	17	2.80E-05	-	4.81E-06	-	Male	Medium			MVYSQASQS	MVYSQASQS			
YIIPRQAPE	F1KPW3;F1KRB6;F1KX94;U1NHN2	F1KPW3	2210	2218	9	5.95E-05	-	9.58E-06	-	Male	High			YIIPRQAPE	YIIPRQAPE			
TYNILPHRQYKV	F1KPQ8	F1KPQ8	1131	1142	12	0.00013494	-	4.22E-06	-	No	Medium		<=WB	ILPHRQYKV	ILPHRQYKV			
LVHPYYSISEQINPGAHFDI	F1L7U1;U1MCN1	F1L7U1	59	78	20	0.00022047	-	3.00E-06	-	No	Medium			PYYSISEQI	VHPYYSISE			
FEVPPIYDSDSATIIAF	U1P3X1	U1P3X1	373	389	17	0.00105082	-	-	-	Male	Medium			IYDSDSATI	IYDSDSATI			
IDTFENTADWGL	F1KW74;U1M1R7	F1KW74	514	525	12	0.00111579	-	-	-	No	Medium			FENTADWGL	FENTADWGL			
ALNGITANDVSSVLT	F1L1C2	F1L1C2	19	33	15	0.00076165	-	-	-	Male	Medium			LNGITANDV	LNGITANDV			
SDPLVFDELTRAQLISDA	F1KQL5;F1KR19	F1KQL5	1520	1537	18	0.00079621	-	-	-	No	High			DELTRAQLI	LVFDELTRA	+		
DSQGNPLAIESVTTNAL	F1KQ47;U1NPX4	F1KQ47	140	156	17	0.00069088	-	-	-	No	Medium			IESVTTNAL	IESVTTNAL			
LGYDVTDNNGILT	F1KPF4;F1KPG3;F1KRA2	F1KPF4	2814	2826	13	0.00070987	-	-	-	Male	Medium			YDVTDNNGI	YDVTDNNGI			
DLGNIEASQAGVAH	F1LCL1	F1LCL1	169	182	14	0.00071196	-	-	-					IEASQAGVA	IEASQAGVA			
WIRSGSLPIDPSWVEADEK	F1KPQ8	F1KPQ8	1822	1840	19	0.00052548	-	-	-	No	Medium			WIRSGSLPI	WIRSGSLPI			
FEQMTISQGLGQYA	F1L4K4;U1NM88	F1L4K4	78	91	14	0.00037086	1.65E-06	-	-	Female	Medium			MTISQGLGQ	MTISQGLGQ	+		
IVESVAQAIGAKQDQ	F1LB74;U1NHZ7	F1LB74	151	165	15	0.00027961	-	-	-					IVESVAQAI	VAQAIGAKQ			
DVPINNFAYINTQKFAM	U1MJ81	U1MJ81	1135	1149	17	0.00026704	-	-	-	Male	Medium	<=WB		FAYINTQKF	FAYINTQKF	+		
GAFYTFSRNHNDIGQIRQD	F1KPW3;F1KRB6;F1KX94	F1KPW3	2068	2086	19	0.00020386	-	-	-	Male	High			YTFSRNHND	FYTFSRNHN	+		
YQYSNANDVQL	F1KQL5;F1KR19	F1KQL5	1375	1385	11	0.00022933	8.48E-07	-	-	No	High			YQYSNANDV	YQYSNANDV	+		
EVITDTGTSFVGGPK	F1L3I0;F1L5L9;F1L723;U1NF90	F1L3I0	280	294	15	0.00038922	1.06E-06	-	-	Male	Medium			ITDTGTSFV	VITDTGTSF			
AQFTPVDDPHL	F1KPJ7;F1KPK1	F1KPJ7	1600	1610	11	0.00040534	-	-	-	No	Low			FTPVDDPHL	FTPVDDPHL			
EITSIEPYLVIH	U1P0K7	U1P0K7	76	87	12	0.00036454	-	-	-	No	Medium	<=WB	<=WB	ITSIEPYLV	ITSIEPYLV			
ALFGEAGVNEIPEITK	U1M2L1	U1M2L1	120	135	16	0.0003333	-	-	-					FGEAGVNEI	LFGEAGVNE			
FLTWSNVPTLSGGGLG	F1L837	F1L837	111	126	16	0.00034559	-	-	-	No	Medium			LTWSNVPTL	FLTWSNVPT			
LSQDFEKFDSNLIANYF	F1KPL9	F1KPL9	2558	2567	17	0.00182564	-	3.61E-06	-	Male	Medium	<=WB		FEKFDSNLI	FEKFDSNLI	+	+	
YQGKKPWALTFSYGRAL	F1L5H2;F1LCU9;U1M5S0	F1L5H2	306	316	17	0.0030714	1.22E-06	-	-	No	High			WALTFSYGR	WALTFSYGR	+		
HMESVHESQTLHLEAQILPIDDNKL	F1KPJ7;F1KPK1	F1KPJ7	133	157	25	0.00365718	-	-	-	No	Low			VHESQTLHL	VHESQTLHL			
WRKEDGSAFGYGVTDSQGVLSF	F1KPF4;F1KPG3;F1KPK5;F1KRA2	F1KPF4	2899	2920	22	0.002091	-	-	-	Male	Medium			YGVTDSQGV	YGVTDSQGV			
WSTAYYDVPIDNFAFTSAQTYRMK	F1L108;U1MJ81	F1L108	48	71	24	0.00147196	-	-	-	Male	Medium	<=SB		FTSAQTYRM	FTSAQTYRM			
WFLGDKELAATETYVF	U1LYA8	U1LYA8	111	120	16	0.0124279	1.07E-06	-	-	No	Medium			LAATETYVF	LAATETYVF	+		
**TINTNVPSDKVPQDFLKKTSALVAK**	**F1KV03;Q6I6Y5;U1MU38**	**F1KV03**	**30**	**49**	**25**	**0.00030632**	**0.0099759**	**-**	**5.04E-05**	**Female**	**Medium**	**<=WB**		**LKKTSALVA**	**FLKKTSALV**	**+**	**+**	**QDFLKKTSALVAKSL**
RTFIAIKPDAVQRGLIGK	F1LG83	F1LG83	54	71	18	2.06E-05	0.0590688	-	-	Female	Medium	<=WB		FIAIKPDAV	FIAIKPDAV	+		
NAGLVVLTDAYLYREPEPPR	F1KQN0	F1KQN0	696	715	20	4.21E-05	0.0001922	-	0.00014	No	Medium			VVLTDAYLY	VVLTDAYLY	+	+	
KNPKLQPYVNAESKIIVDDVK	U1M695;U1MUX9	U1M695	44	64	21	0.00011558	1.71E-05	2.92E-06	0.00041	No	High	<=WB		YVNAESKII	LQPYVNAES	+		
DQSGEFMFDFDGDEIFH	DRA*0101	DRA*0101	17	33	17	0.000317	8.37E-06	1.69E-05	4.04E-05					FDFDGDEIF	MFDFDGDEI	+		
YSRIGSTLNIRIGPEGRYEPPLN	F1KPW3;F1KRB6;F1KX94	F1KPW3	476	498	23	0.00039987	6.05E-05	1.08E-05	1.04E-05	Male	High			IGSTLNIRI	IGSTLNIRI	+		
TNWWLPVPDTGFHATK	U1MI86	U1MI86	58	67	16	0.00296395	0.0007147	1.02E-05	8.15E-06	Male	Medium			WLPVPDTGF	PVPDTGFHA			
LPVPDTGFHATKIRVF	U1MI86	U1MI86	58	67	16	0.00296395	0.0007147	1.02E-05	8.15E-06	Male	Medium	<=WB		FHATKIRVF	FHATKIRVF	+		
TSSDEISKHISGVIMFHE	F1L5H2;F1LCU9;U1M5S0	F1L5H2	77	93	18	0.00193648	0.0003789	1.53E-05	5.56E-05	No	High			ISKHISGVI	ISKHISGVI	+		
LTAKQNEYVQVFGASNVFDGSAIK	U1MRA1	U1MRA1	669	692	24	0.00020175	2.57E-05	0.0002	-	No	High			YVQVFGASN	VQVFGASNV	+		
**YENQFAWNKVGNVLF**	**F1KPY0**	**F1KPY0**	**949**	**963**	**15**	**0.00290505**	**0.0002515**	**0.00327**	**-**	**Male**	**Medium**	**<=WB**		**FAWNKVGNV**	**FAWNKVGNV**	**+**		**ENQFAWNKVGNVLFI**
ALDGLAQNVANAFKNYDKPS	U1MRA1	U1MRA1	698	717	20	0.00111059	0.000334	0.00062	8.84E-06	No	High			LAQNVANAF	LAQNVANAF	+		
VSQPNEGYQLSSANKLFVKE	F1L1S5	F1L1S5	108	123	20	0.0006417	0.0008592	1.04E-05	-	No	High	<=SB	<=WB	YQLSSANKL	YQLSSANKL	+	+	
NQFAWNKVGNVLFIESPRDVG	F1KPY0	F1KPY0	951	969	21	0.00247919	0.0002385	1.24E-05	-	Male	Medium	<=WB		FAWNKVGNV	FAWNKVGNV			
PHYTTFDGQVYE	F1KUR2;U1MQK3	F1KUR2	156	167	12	0.00145242	0.0001807	1.07E-05	-	No	Medium			YTTFDGQVY	YTTFDGQVY	+		
FAEVERYDTKLTPTHIVVL	U1MRA1	U1MRA1	383	396	19	0.0026868	0.0008279	9.64E-05	-	No	High			TKLTPTHIV	VERYDTKLT			
ALHWDFENSRSSAFARVE	F1KQF7;U1NL82	F1KQF7	516	533	18	0.00062861	0.000956	-	-	No	Medium			FENSRSSAF	FENSRSSAF	+		
ISVPGGGVSKAVYFPIVPTQIGEVK	F1KQN0;F1KRK7	F1KQN0	902	922	25	0.00046595	0.0004894	-	-	No	Medium	<=WB		YFPIVPTQI	YFPIVPTQI	+		
ETEKDSFIKDVLPVEL	F1L8V4;U1MKZ2	F1L8V4	70	85	16	0.00017016	0.001689	-	-	No	Medium			IKDVLPVEL	SFIKDVLPV	+		
YVVGPDRFITFDGRVF	F1KPM2;U1NWH3	F1KPM2	2607	2622	16	7.48E-06	0.0002956	-	-	Female	Medium			FITFDGRVF	FITFDGRVF	+		
VHGVMQTSSAFLPHRE	U1P2P9	U1P2P9	76	91	16	1.56E-05	0.0010085	-	-	No	Medium	<=WB		VMQTSSAFL	VMQTSSAFL	+		
VKSSNNSNYTVTPNMGIVE	F1LGP8	F1LGP8	38	56	19	6.16E-05	0.000597	-	-			<=WB		YTVTPNMGI	YTVTPNMGI	+		
HDDNEQIVQSGAILRH	F1L8V4;U1MKZ2	F1L8V4	14	29	16	5.37E-05	0.0006675	-	-	No	Medium			IVQSGAILR	IVQSGAILR	+		
LHDDNEQIVQSGAILR	F1L8V4;U1MKZ2	F1L8V4	14	29	16	5.37E-05	0.0006675	-	-	No	Medium			IVQSGAILR	IVQSGAILR			
FDVGLALTEAKNILNR	F1L4K4;U1NM88	F1L4K4	143	154	16	0.0213562	1.94E-05	-	-	Female	Medium	<=WB		LALTEAKNI	LALTEAKNI	+		
SSLSMTKDGLIE	F1L0R7	F1L0R7	195	206	12	0.00458749	1.03E-05	-	-	No	Medium			LSMTKDGLI	LSMTKDGLI	+		
NELSYKMAHVSTVSE	F1KUR2;U1MQK3	F1KUR2	199	211	15	0.00040823	9.68E-05	-	-	No	Medium	<=SB		YKMAHVSTV	YKMAHVSTV	+		
YGTDEIDGDYWIVKNSWGPGWGEKG	F1L0U9;F1L4M4;Q8MTV5;U1NPZ8	F1L0U9	76	100	25	0.00034456	0.0001582	-	-	No	Medium			YWIVKNSWG	VKNSWGPGW	+		
AHYLQWNGNPLS	F1KUR2;U1MQK3	F1KUR2	832	843	12	0.0002724	5.86E-05	-	-	No	Medium			YLQWNGNPL	YLQWNGNPL	+		
LQRTVPVAVPGIVF	F1L5H2;F1LCU9;U1M5S0	F1L5H2	267	280	14	0.00067841	1.13E-05	-	-	No	High			PVAVPGIVF	PVAVPGIVF	+		
VYCSHGSGSLVLQLMKCLS	U1M5B0	U1M5B0	63	80	19	0.0014195	1.49E-05	-	-					YCSHGSGSL	LVLQLMKCL	+		
VENIIESEGNKF	F1KPM2;U1NWH3	F1KPM2	1905	1916	12	0.00116674	0.000222	-	-	Female	Medium			IIESEGNKF	IIESEGNKF	+		
GESYAGVYTPTLTDLLIKR	F1KPY0	F1KPY0	1018	1034	19	0.00351199	0.0001313	-	-					YTPTLTDLL	YTPTLTDLL	+		

Consensus peptide sequence is provided in the first column followed by the protein sources. Starting and end position in the lead protein from which the peptide derives is also shown. The peptide size and log2 transformed summed intensity of the different experiments is shown as well. The relative enrichment of the protein source (Female for ESF, Male for ESM or "No" if it is not found enriched) as well as the total abundance estimated by the emPAI are indicated. Potential binders (SB for strong binding; WB for weak binding) for each peptide and MHCII allotype are indicated. For consensus peptides not found in one condition summed intensities are shown as "-". For each consensus peptide the NetMHCIIPan binding core for each allotype is also indicated. Finally, the IEDB_I column indicates whether a peptide was predicted to be immunogenic by the IEBD tool, and the Tested peptide column indicates the peptide assayed for T cell reactivity after expansion with either ESF or ESM. We additionally included two peptides as negative controls: F1LEI7$_{25\text{-}44}$ (PVSMSTIPEEYKEFVPEVVQNFYKD) and F1KSI5$_{23\text{-}47}$ (RMATCVSLKPISDVDFLTATVDKKN).
